# Cardiomyocyte infection by *Trypanosoma cruzi* promotes innate immune response and glycolysis activation

**DOI:** 10.3389/fcimb.2023.1098457

**Published:** 2023-02-06

**Authors:** Gabriela Venturini, Juliana M. Alvim, Kallyandra Padilha, Christopher N. Toepfer, Joshua M. Gorham, Lauren K. Wasson, Diogo Biagi, Sergio Schenkman, Valdemir M. Carvalho, Jessica S. Salgueiro, Karina H. M. Cardozo, Jose E. Krieger, Alexandre C. Pereira, Jonathan G. Seidman, Christine E. Seidman

**Affiliations:** ^1^ Department of Genetics, Harvard Medical School, Boston, MA, United States; ^2^ Laboratory of Genetics and Molecular Cardiology, University of São Paulo Medical School, São Paulo, Brazil; ^3^ Division of Cardiovascular Medicine, Radcliffe Department of Medicine, University of Oxford, Oxford, United Kingdom; ^4^ Wellcome Centre for Human Genetics, University of Oxford, Oxford, United Kingdom; ^5^ LizarBio Therapeutics, Sao Paulo, Brazil; ^6^ Department of Microbiology, Immunology and Parasitology, Escola Paulista de Medicina, São Paulo, Brazil; ^7^ Division of Research and Development, Fleury Group, São Paulo, SP, Brazil; ^8^ Division of Cardiovascular Medicine, Department of Medicine, Brigham and Women’s Hospital, Boston, MA, United States; ^9^ Howard Hughes Medical Institute, Chevy Chase, MD, United States

**Keywords:** Chagas cardiomyopathy, *Trypanosoma cruzi*, metabolism, inflammation, glycolysis

## Abstract

**Introduction:**

Chagas cardiomyopathy, a disease caused by *Trypanosoma cruzi* (*T. cruzi*) infection, is a major contributor to heart failure in Latin America. There are significant gaps in our understanding of the mechanism for infection of human cardiomyocytes, the pathways activated during the acute phase of the disease, and the molecular changes that lead to the progression of cardiomyopathy.

**Methods:**

To investigate the effects of *T. cruzi* on human cardiomyocytes during infection, we infected induced pluripotent stem cell-derived cardiomyocytes (iPSC-CM) with the parasite and analyzed cellular, molecular, and metabolic responses at 3 hours, 24 hours, and 48 hours post infection (hpi) using transcriptomics (RNAseq), proteomics (LC-MS), and metabolomics (GC-MS and Seahorse) analyses.

**Results:**

Analyses of multiomic data revealed that cardiomyocyte infection caused a rapid increase in genes and proteins related to activation innate and adaptive immune systems and pathways, including alpha and gamma interferons, HIF-1α signaling, and glycolysis. These responses resemble prototypic responses observed in pathogen-activated immune cells. Infection also caused an activation of glycolysis that was dependent on HIF-1α signaling. Using gene editing and pharmacological inhibitors, we found that *T. cruzi* uptake was mediated in part by the glucose-facilitated transporter GLUT4 and that the attenuation of glycolysis, HIF-1α activation, or GLUT4 expression decreased *T. cruzi* infection. In contrast, pre-activation of pro-inflammatory immune responses with LPS resulted in increased infection rates.

**Conclusion:**

These findings suggest that *T. cruzi* exploits a HIF-1α-dependent, cardiomyocyte-intrinsic stress-response activation of glycolysis to promote intracellular infection and replication. These chronic immuno-metabolic responses by cardiomyocytes promote dysfunction, cell death, and the emergence of cardiomyopathy.

## 1 Introduction

Heart failure is a global epidemic that affects 38 million patients worldwide and is the result of multiple underlying cardiovascular and systemic disorders (Heidenreich et al., 2013; Roger, 2013; Braunwald, 2015; Benjamin et al., 2018). Infectious diseases and their associated inflammatory responses are substantial causes of heart failure in low and low-middle income countries where the majority of the world lives. In Latin America, *Trypanosoma cruzi* (*T. cruzi*), an endemic parasite that causes Chagas cardiomyopathy, is the most common infectious cause of heart failure. The World Health Organization (WHO) estimates that across the Americas, Chagas disease has a global disease burden (disability-adjusted life-years) that is 7.5-fold greater than malaria (Mathers et al., 2018). Chagas disease is not restricted to Latin America, and in 2016, analyses estimated that 240,000 U.S. citizens and an additional 100,000 undocumented residents had *T. cruzi* infection, accounting for 30,000-45,000 Chagas cardiomyopathy cases and 60-300 congenital infections that annually occur in this country (Manne-Goehler et al., 2016; Bocchi et al., 2017). California, Florida, New York, and Texas each had over 10,000 cases/year (Manne-Goehler et al., 2016).

Acute *T. cruzi* infection causes nonspecific and mild symptoms that often escape clinical attention and promotes unrecognized life-long chronic infection (Maguire, 2006). Approximately 20-30% of asymptomatic, but chronically infected individuals, develop cardiomyopathy, which emerges years or decades after the initial or repeated infections (Costa et al., 2017). Chagas cardiomyopathy has no tailored treatment options and management mainly addresses symptom relief (Martinez et al., 2020).

There are considerable gaps in understanding Chagas cardiomyopathy pathogenesis (Bonney et al., 2019). *T. cruzi* infects and elicits a rapid immune response in most tissues that clears the parasite. However, case reports demonstrate myocardial reactivation of viable parasites with immunosuppression, implying that the heart, similar to the gastrointestinal system, can provide a cellular reservoir for the parasite (Burgos et al., 2010; Nagajyothi et al., 2012; Lewis and Kelly, 2016; Ward et al., 2020). The mechanisms by which *T. cruzi* lays dormant in the heart and evades the immune response is unknown. However, the consequences are readily identified, as chronic host-pathogen interactions evoke cardiac remodeling and the emergence of heart failure decades after acute infection (Higuchi et al., 2003; Machado et al., 2012; Pérez-Mazliah et al., 2021).

Human iPSC-derived cardiomyocytes (iPSC-CMs) infected with *T. cruzi* have provided some insights into host-pathogen interactions. Previous analyses of these cellular models demonstrate increased reactive oxidant species (ROS), mammalian target of rapamycin (mTOR)-activation of oxidative phosphorylation, and production of inflammatory cytokines that are predicted to promote immune cell migration (Dias et al., 2017; Libisch et al., 2018; Bozzi et al., 2019). However, the processes that trigger and integrate these diverse and fundamental changes in cardiomyocyte biology are unknown. Moreover, whether these changes in cardiomyocytes are adaptative and protective or detrimental to parasite or cardiomyocyte survival is unknown.

We exploited the iPSC-CMs model of *T. cruzi* infection to discern genome-wide transcriptional responses to *T. cruzi* infection and validated these with proteomic and metabolic analyses. Our studies uncover highly integrated immuno-metabolic responses that are intrinsic to *T. cruzi*-infected cardiomyocytes and closely resemble prototypic responses in pathogen-activated immune cells. Using pharmacological and genetic perturbations of key signaling molecules we demonstrate that the parasite capitalizes on these responses to augment infection and increase intracellular replication. Together these data demonstrate that a highly conserved innate immune response and metabolic rewiring occurs in cardiomyocytes that is used by the parasite to promote intracellular infection and replication and evokes profound changes in cardiomyocyte physiology and function.

## 2 Materials and methods

### 2.1 Isolation and maintenance of *T. cruzi* Y-strain trypomastigotes

*T. cruzi* trypomastigotes (Y strain) and GFP-Y strain (Ramirez et al., 2000) (kindly provided by Dr. Sergio Schenkman lab) were derived from the supernatants of infected LLC-MK2 culture cells grown. Briefly, sub-confluent cultures of LLC-MK2 cells were infected with 5x10^6^ trypomastigotes. Free parasites were removed after 24 hours, and the cell cultures were maintained in 2% FBS-RPMI 1640. Five days following infection, free trypomastigote forms were found in the cell supernatants. Parasite containing supernatant were collected, centrifuged at 2000xg for 20min, resuspended in RPMI plus B27 supplement, counted in a Neubauer Chamber and added to the cells in the desired MOI.

### 2.2 Human induced pluripotent stem cells derived cardiomyocyte (iPSC-CM) infection protocol

iPSC-CMs were differentiated as previously described (Sharma et al., 2018a). All experiments were carried out between day 30-35 of differentiation. iPSC-CMs were infected with purified trypomastigotes in RPMI media plus B27 in a MOI of 1 (number parasite/number cardiomyocyte) for omics experiments; and MOI 5 for image experiments. Media was changed after 24 hours to remove free parasites. All experiments were performed with 3 independent iPSC-CM differentiation and infection.

### 2.3 RNA-sequencing analysis

RNA-sequencing analysis were performed as described (Sharma et al., 2020) with few modifications. Briefly, after Trizol RNA extraction, libraries were prepared using the Nextera library preparation method. RNA-Seq library samples were pooled and ran on the Illumina NextSeq500 platform. Sequenced reads were aligned with STAR (Engström et al., 2013). Read counts per gene were normalized using DEseq2 and used for group comparison (Love et al., 2014). Gene pathway enrichment analysis were conducted using clusterProfiler (Yu et al., 2012) using the Hallmark Database Gene Set as reference (Liberzon et al., 2015).

### 2.4 Proteomics analysis

After 48 hours, non-infected and infected cells (1x10^6^ cells) were scrapped with 300 μL of protein extraction buffer (8M urea and 50 mM of ammonium bicarbonate). Digestion and mass spectrometry methods were performed as described (Malagrino et al., 2017) with few modifications. Briefly, total protein (100 μg) extracts were treated with 2.5 μL of 100 mM DTT at 60°C for 30 minutes and alkylated with 2.5 μL of 300 mM iodoacetamide for 30 minutes at room temperature in the dark. Proteins were then enzymatically digested with 10 μL of trypsin 0.05 μg/μL for 16 hours at 37°C. To stop the digestion, we added 10 μL of 5% trifluoroacetic acid (TFA). Tryptic peptide solution was centrifuged at 16,000xg for 30 minutes at 6°C and peptides were desalted with zip-tip C18.

### 2.5 Metabolomics analysis

Metabolomic analyses were performed as described (Venturini et al., 2019), with few modifications. After 3, 24 and 48 hours of infection, 1x10^6^ cells were washed with cold PBS and scrapped with 1 mL of cold Acetonitrile:Isopropanol:MilliQ water (3:3:2 v/v/v) for metabolite extraction. Metabolites were centrifuged at 15800xg at 0°C for 5 minutes. An aliquot of supernatant (900 μL) was transferred to a new tube and both, protein pellet and supernatant, were dried during 18 hours in speed-vac. The metabolites were resuspended in 1 mL of cold Acetonitrile:MilliQ water (1:1 vol/vol), centrifuged at 15800xg at 0°C for 5 minutes and 900 μL of supernatant were transferred to a new tube. 5 μL of myristic acid D27 (3 mg/mL) was added as internal standard and retention time index and the solution was dried for 18 hours. Metabolites were derivatized with 20 μL of methoxylamine diluted in pyridine (40 mg/mL) for 16 hours at room temperature, following addition of 90 μL of MSTFA with 1% of TMCS and one hour of incubation at room temperature. Metabolites were centrifuged at 15800xg at 0°C for 5 minutes and 100 μL of supernatant were transferred to a glass insert. After derivatization, 1 µL of this derivative was used for Gas Chromatography Mass Spectrometry (GC/MS) analysis.

### 2.6 Extracellular metabolic flux analysis

Glucose and lactate in media were measured using an electrolyte counter (FLEX ABL800 Radiometer Medical, Bronsho, Denmark). SeaHorse XF Mito Stress Assay and SeaHorse XF Glycolytic Rate Assay (Agilent Technologies) were performed according to manufacturer’s instruction in a 96 wells Seahorse XF. Briefly, iPSC-CM were plated in 96 well seahorse plates coated with matrigel 5 days before the experiment. Seahorse XF Base Medium (XBMS) was supplemented with 1 mM sodium pyruvate, 2 mM glutamine, and 10 mM glucose and set up to pH 7.6 at 37°C. iPSC-CM were rinsed with XBMS and incubated with 180 μL at 37°C without CO_2_ for one hour. Mitochondrial chain or glycolytic pathway inhibitors from the Seahorse XF Cell Mito Stress Test (Agilent Technologies) were resuspended in XMBS following manufacturer protocol, then diluted to 10x final concentrations and loaded into the sensor cartridge ports for injection. Final concentrations were 1 μM oligomycin (ATP synthase inhibitor), 2.5 μM FCCP (which uncouples ETC activity from ATP synthesis), 1 μM each of rotenone and antimycin A (R/A) (ETC complex I and III inhibitors) and 50 mM of 2DG (Hexokinase inhibitor). The Seahorse XF96 was programed to mix for 3 minutes, wait 2 minutes then obtain measurements for 3 minutes. This cycle was repeated 3 times after each drug injection. Basal respiration was calculated as the difference between OCR at baseline and that after R/A injection. Spare respiratory capacity (SRC) was calculated as the difference between OCR after FCCP injection and that at baseline. Data were normalized by total protein measured using BCA assay.

### 2.7 Drug treatments

All drug treatment experiments were carried out with a MOI of 5. iPSC-CMs were pre-treated 16 hours before the infection with drugs and maintained over 48 hours of infection (unless a different condition is explicated in the Results section). Twenty-four hours after infection, media was changed by fresh media containing the drug to remove free parasites. Controls were performed using media or the vehicle. Drugs used in this are described in **Supplementary Material Table 1**.

### 2.8 HIF-1α mutagenesis using CRISPR technology

CRISPR clones were developed as previously described (Sharma et al., 2018b; Sharma et al., 2020). Briefly, gRNA sequences were designed using CRISPR design tool (https://portals.broadinstitute.org/gpp/public/analysis-tools/sgrna-design). gRNAs were cloned in a plasmid using Zero Blunt TOPO PCR Cloning Kit (Thermo Scientific), and isolated colonies were purified using MiniPrep (Quiagen). PGP1 cells (Lee et al., 2009) were transfected with 40 µM of Cas-9 plasmid (pSpCas9(BB)-2A-Puro V2.0 (PX459). (Addgene plasmid # 62988) and 25 μM of sgRNA using AMAXA Nucleofactor (Lonza). At 48 hours post transfection, cells were selected with puromycin (1 µg/mL). Selected cells replated in low confluence to allow single cell colony growth. Isolated colonies were picked and sequenced by Sanger and MiSeq methods to confirm editing. Subcloning was carried out, as necessary to ensure that a clone contained one genotype. Two clones HIF-1α^Δ301/-^ and HIF-1α^Δ301-305/+^ (**Supplemental Figure 9D**) were selected for cardiomyocytes differentiation and subsequent studies.

HIF-1α gRNA sequence:

TGTACAAAAAAGCAGGCTTTAAAGGAACCAATTCAGTCGACTGGATCCGGTACCAAGGTCGGGCAGGAAGAGGGCCTATTTCCCATGATTCCTTCATATTTGCATATACGATACAAGGCTGTTAGAGAGATAATTAGAATTAATTTGACTGTAAACACAAAGATATTAGTACAAAATACGTGACGTAGAAAGTAATAATTTCTTGGGTAGTTTGCAGTTTTAAAATTATGTTTTAAAATGGACTATCATATGCTTACCGTAACTTGAAAGTATTTCGATTTCTTGGCTTTATATATCTTGTGGAAAGGACGAAACACCGCTAAAGGACAAGTCACCACGTTTTAGAGCTAGAAATAGCAAGTTAAAATAAGGCTAGTCCGTTATCAACTTGAAAAAGTGGCACCGAGTCGGTGCTTTTTTTCTAGACCCAGCTTTCTTGTACAAAGTTGGCATTA

Primer’s sequence:

Forward – GTTTTCCAAAACAATGATGAACA

Reverse – TGAGAAATAAACATTTTTGGGGA

### 2.9 Lentivirus

Lentivirus particles for GLUT4 silencing were produced using SLC2A4 – GLUT4 Human shRNA Plasmid Kit (Sigma) according to manufacturer instructions. iPSC-CM were transduced with GLUT4 or scramble particles over 48 hours prior to *T. cruzi* infection. Cell contractility after GLUT4 silencing was measured using SarcTrack (Toepfer et al., 2019).

### 2.10 High content screening analysis

4x10^4^ iPSC-CM cells were plated in a 96 wells plate for microscopy analysis. After drug treatment and infection, cells were washed with PBS and fixed with 4% PFA diluted in PBS for 30 minutes. Cells were blocked for one hour with 2% casein and incubated for 3 hours with phalloidin conjugated with Alexa 555 for actin staining and DAPI for nuclei and parasite staining. Cells were washed and kept in PBS until analysis. Images were acquired in a high content screening microscope IN Cell Analyzer 2200 (GE Life Science) and Arrayscan xTi (Thermo Scientific) with 12 photos per well in 400x magnification, 5 wells per group. The number of cells and parasites were counted using a MATLAB script specifically developed for this procedure.

## 3 Results

### 3.1 Transcriptomic and proteomic profiles of *T. cruzi* infected iPSC-CMs

**Figure 1:**
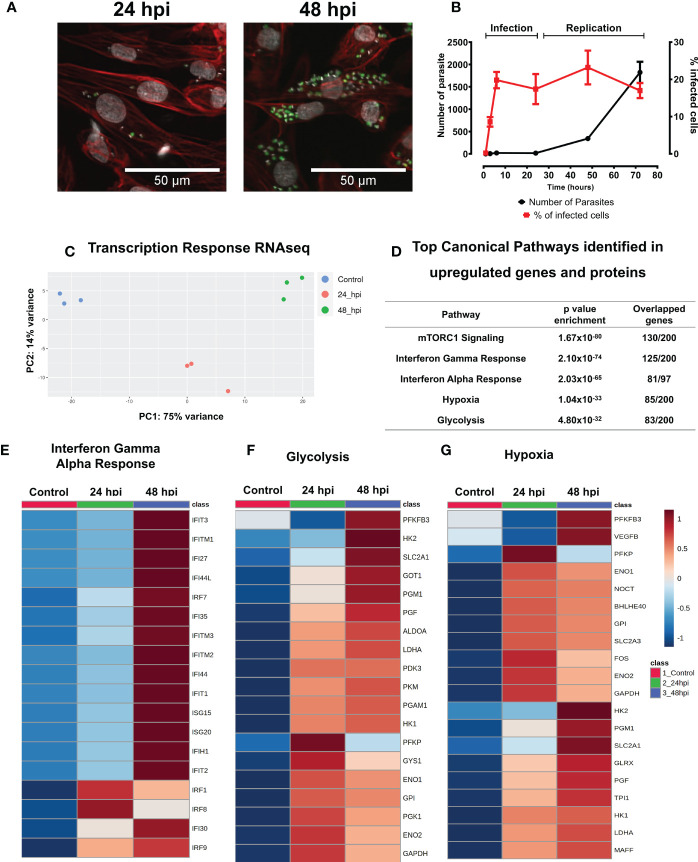
*T. cruzi* infected IPSC-CMs activate immune responses. **(A)** With a multiplicity of infection (MOI) 5:1 (parasite:cell), 20% of iPSC-CMs contain parasites at 24 hours that multiply 4-fold by 48 hpi, and lyse cells by 72 hpi. Fluorescent images are at 24 hpi and 48 hpi detect GFP-tagged *T. cruzi* (green), phalloidin-strained actin (red) and DAPI-stained nuclei (grey). **(B)** Percentage of infected iPSC-CMs and parasite numbers during infection and replication phases. **(C)** Transcriptomic profiles of iPSC-CMs displayed by principal component analyses. Control, uninfected (blue), 24 hpi (red) and 48 hpi (green) showing overall difference among groups in a PCA plot. **(D)** Top Canonical Pathways enriched by both, genes and proteins, upregulated after 24 hpi and 48 hpi. **(E–G)**. Heatmaps of differentially expressed genes involved in glucose metabolism, HIF-1α signaling, and interferon signaling.

We characterized the infection cycle of *T. cruzi* trypomastigotes in 30-day old iPSC-CMs. Parasites were inoculated (MOI = 5) resulting in infection of approximately 20% of exposed iPSC-CMs at 6 hours post infection (hpi). Intracellular parasite replication occurred between 24 and 48 hpi and infected iPSC-CMs burst after 72 hpi (**Figures 1A, B**, and **Figures S1A, S1B**). A similar infection cycle was observed in rat neonatal cardiomyocytes (**Figure S1C**).

We performed and compared RNA-seq of control iPSC-CMs (unexposed to parasite) and infected iPSC-CM cultures at 24 hpi and 48 hpi (**Figure 1C** and **Tables S1–S3**).

The infection of iPSC-CM *T. cruzi* resulted in many transcriptional changes, as represented in the PCA plot (**Figure 1C**), and detailed described in Supplementary Tables (**Tables S4–S7**). We identified an abrupt decrease in expression of genes participants of contractile apparatus, such as sarcomere and cytoskeleton proteins (**Figure S2**, **Tables S6, S7**). In comparison to controls, infected iPSC-CMs had decreased transcripts levels of key contractile proteins including actin and actinin isoforms *(ACTA1, ACTN2)*, myosin (*MYH7, MYH11, MYH9*), myosin associated light chains *(MYL7, MYL2)*, and troponins *(TNNT2, TNNC2)*.

By contrast, infection caused a marked enrichment of molecular pathways associated with immune/inflammatory activation. Infected cells had increased expression of several C-type lectin receptors and toll-like receptor (TLRs) genes that recognize pathogen-associated molecular patterns, including *CLEC17A, TLR3, TLR5* and the *TLR* adaptor protein *MYD88* (**Tables S2, S5**). TRIM genes that encode tripartite motif proteins, and TRIM-target genes (*DDX58*; retinoic acid inducible gene-1) that evoke downstream TLRs signals, were also increased. Notably, infected iPSC-CMs had increased expression of *HELZ2* and *PHF11*, which encode molecules that enable persistent expression of TLR-induced immune activation. In addition, and in agreement with previous data in literature, infected iPSC-CMs also had signatures associated with markedly increased mTOR activation, being the top enriched canonical pathway identified in our data (**Figure 1D**).

RNA-seq data also showed enhanced interferon-mediated signaling (**Figures 1D, 1E**), including interferon induced genes *(IFI27, IFI30, IFI35, IFI44, IFIT1, IFIT2, IFIT3, IFITM1, IFITM2, IFITM3, IFIH1, IFR1, IFR7, IFR8, IFR9, ISG15, ISG20)*, chemoattractant molecules and chemokine receptors *(CCL2, CCL5, CCL21, IL6R, IL1R, CXCL10, CXCL11)*, and cytokines *(IL1B, IL6, IL16, IL15)*.

**Figure 2:**
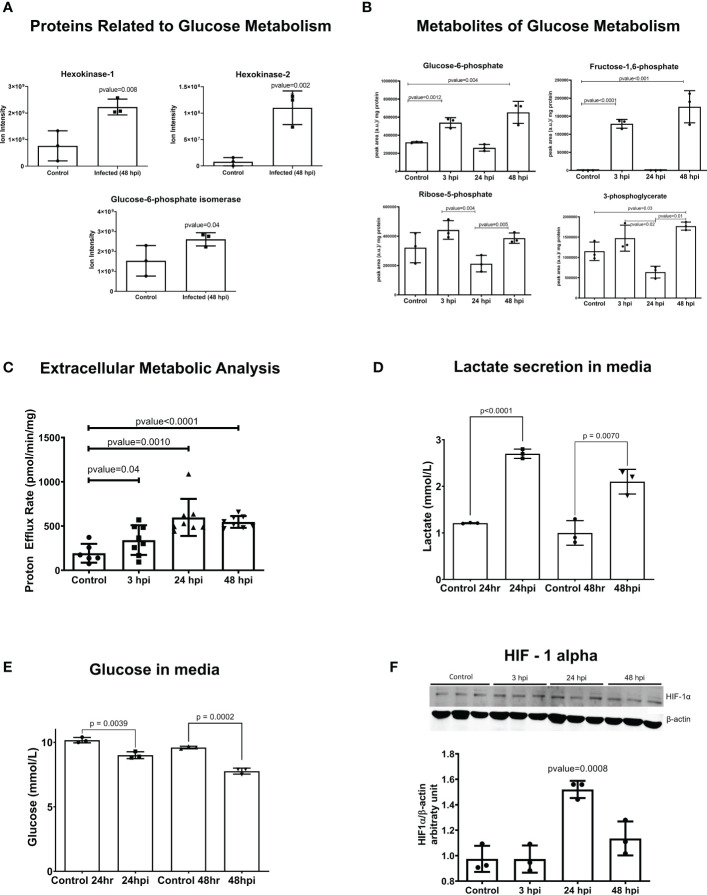
Glycolysis is activated in iPSC-CMs after *T. cruzi* infection. **(A)** Mass spectrometry (Ion Intensity) demonstrate increased levels of key metabolic regulatory enzymes (hexokinase 1 (HK1), hexokinase 2 (HK2) and glucose-6-phosphate isomerase (GPI). **(B)** Intermediate metabolites of glycolysis are increases at 3 hpi, demonstrating an early glycolytic switch after infection. **(C)** Extracellular metabolic flux analysis shows proton efflux rate in infected IPSC-CMs. **(D)** Lactate secreted in the media after parasite infection. **(E)** Glucose concentration in media showing the consumption after parasite infection. **(F)** HIF-1α protein is increased in iPSC-CMs at 24 hpi, consistent with transcriptomic data. Data reflect three independent replicates for each experiment. Statistical analyses used ANOVA test with Bonferroni correction or t*-*test when appropriate and p-value<0.05 was considered significant.

Glycolysis was identified as another canonical pathway that was upregulated in transcriptomic data at 24 hpi and 48 hpi (**Figures 1D, 1F**, **2A**, and **Tables S4, S5**). We observed upregulation of key enzyme genes in this metabolic pathway, including *HK2* and *HK1, LDHA, PFK*, and *PGM*.

Hypoxia was also a highly enriched gene pathway identified by RNA-seq analyses (**Figure 1D, 1G** and **Tables S4, S5**). Activation of HIF-1α signaling is a common phenomenon to many human infections including bacterial, viral and intracellular parasites such as *Leishmania amazonensis* and *Toxoplasma gondii*. HIF-1α levels were increased in iPSC-CMs at 24 hpi (**Figure 2F**), possibly due to increased phosphorylation of ERK (**Figure S4B**) or AKT-mTOR signaling that can augment HIF-1α translation (Semenza, 2003). Levels of transcripts encoding prolyl hydroxylases *(EGLN2)* and von Hippel Lindau protein *(VHL)* that promote HIF-1α degradation were unchanged in infected compared to control iPSC-CMs. Moreover, RNA-seq data showed increased expression of multiple direct gene targets of HIF-1α activation. At 48 hpi, transcripts encoding proteins that regulate glucose metabolism *(HK1, HK2, GPI, ENO1, ENO2, GAPDH, LDHA, PFKBP3, PFKP, PGM1, PGK1, TPI* and *GLUT1 (SLC2A1))* and *VEGFB* were significantly increased (**Figure 1F** and **Tables S2, S5**).

The expression of Interferon alpha/gamma (IFN) pathway-related genes was significantly and positively correlated with hypoxia and glycolysis pathway-related genes (as described in **Tables S15, S16**, with R values greater than 0.7 and FDR-corrected p-values less than 0.005). Specifically, 93 IFN pathway genes positively correlated with 27 hypoxia pathway genes and 20 glycolysis pathway genes and increased over the course of infection (0 hpi < 24 hpi < 48 hpi). Seventy-two (72) IFN pathway genes, 4 hypoxia pathway genes, and 8 glycolysis genes increased only at 48 hpi and were positively correlated (0 hpi = 24 hpi < 48 hpi); and 7 IFN pathway genes, 6 hypoxia pathway genes, and 3 glycolysis pathway genes increased only at 24 hpi and were positively correlated (0 hpi = 48 hpi < 24 hpi).

Oxidative phosphorylation was a pathway slightly enriched at 24 hpi, but highly increased at 48 hpi, concomitant to glycolysis (**Tables S4, S5**).

We confirmed our findings from transcriptional data using shotgun proteomics (**Figure S3**, **Tables S10, S11**). At 48 hpi, we observed high enrichment in the interferon-gamma-mediated signaling pathway, as well as the expression of transcriptional regulators for interferons *(IRF1, DTX3L)* and molecules activated by interferons *(HERC5, ISG15, MX1)*. We also identified a similar profile in glycolytic enzymes and TCA-related genes.

Our comprehensive analysis of transcriptomic and proteomic data reveals that *T. cruzi* infection in iPSC-CMs stimulates a robust immune response, characterized by the activation of the mTOR-HIF-1α signaling pathway and glycolytic metabolism. Notably, these same pathogen-induced signals are also observed in monocytes and macrophages, where they stimulate the activation of glycolysis. This suggests that *T. cruzi* infection may exploit common immune and metabolic pathways to facilitate its own replication and spread within host cells.

### 3.2 Metabolic rewiring during *T. cruzi* infection

The glycolytic activation occurs in pathogen-activated macrophages, perturbing the mitochondrial tricarboxylic acid (TCA) cycle so as to increase succinate, a proinflammatory metabolite that enhances HIF-1α activity (Tannahill et al., 2013; Mills and O’Neill, 2016; Mills et al., 2016). Given evidence for both increased HIF-1α activity and a glycolytic activation in *T. cruzi*-infected iPSC-CMs, we performed metabolomic and extracellular metabolic flux analyses to profile the glycolytic metabolism (**Figures 2B, C**) and TCA cycle (**Figures S6, S7**).

Metabolomic data, consistent with RNA-seq and proteomic data, showed upregulation of glycolysis intermediates after infection. These metabolites increased from the beginning of infection at 3 hpi and remained upregulated at 48 hpi. However, it is worth noting that the dynamic nature of metabolism means that the production-to-consumption ratio of a metabolite may not always be accurately captured using an unlabeled metabolomic approach. This could explain why we observed decreased glycolytic intermediates at 24 hpi.

Our extracellular metabolic analysis (**Figure 2C**) demonstrated an increase in proton efflux rate starting at 3 hpi, which was sustained until 48 hpi, consistent with the findings from our metabolomics data. Additionally, we observed significant differences in glucose consumption and lactate secretion in the media after infection (**Figures 2D, E**). Analyses throughout the infection cycle showed transiently increased levels of citrate at 3 hpi and sustained increased levels of succinate through 48 hpi. Progression through the TCA cycle should normally increase fumarate and malate as a downstream consequence of increased succinate levels. However, this was not observed (**Figure S6**) and instead our data suggest an unbalance in the TCA cycle that sustains increased succinate levels. Additionally, RNA-seq data indicated increased expression of succinate dehydrogenases (SDHB; **Table S2**) which increases mitochondrial oxidation of succinate and ROS production. We also observed increased catabolism of several amino acids (glutamine, isoleucine, threonine, and valine) in iPSC-CMs after 24 hpi, indicating that intense anaplerosis likely contributes to TCA production of succinate, as was previously observed in macrophages (Corcoran and O’Neill, 2016).

Infected iPSC-CMs had increased oxygen consumption rate (OCR), and maximally increased mitochondrial respiration was observed at 48 hpi (**Figure S6B**). Extracellular flux experiments showed incomplete coupling of ATP production with oxygen consumption, which is indicative of mitochondrial proton leak (**Figure S5A**). In addition, increased secretion of nitric oxide was identified in the culture media after 24 hpi (**Figure S5B**). These abnormalities would increase ROS levels, which is known to improve *T. cruzi* replication in macrophages. In concordance with increased OCR, we identified genes and proteins from oxidative phosphorylation upregulated at 48 hpi (**Tables S5**, **S11**) and 498 upregulated genes (**Table S2**) related to mitochondria metabolism, structure, transporters/channels, and dynamics based on the MitoCarta Inventory (Rath et al., 2020).

Altogether, metabolomics analysis showed activation of glycolysis since the first hours of infection remaining up to 48 hpi; an unbalance of TCA, with upregulation of succinate that was not followed by fumarate and malate, amino acid anaplerosis and increased OCR after infection.

### 3.3 Manipulation of immuno-metabolic signals in *T. cruzi* infected-iPSC-CMs

We considered whether attenuating components of immuno-metabolic signals identified in *T. cruzi*-infected iPSC-CMs would influence susceptibility to parasite infection and/or parasite replication. We assessed parasite infectivity by quantifying the numbers of intracellular *T. cruzi* per total number of iPSC-CMs at 24 hpi. The same assessment at 48 hpi was used to index parasite replication rates.

First, we tested a multi-faceted polyphenolic compound, resveratrol. Pre-treatment (16 hours prior to parasite inoculation) and chronic supplementation with resveratrol to iPSC-CMs and neonatal rat cardiomyocytes (**Figures S5C, S5D**) did not alter parasite infection but significantly depressed amastigote proliferation rates (**Figures S5C–S5E**).

**Figure 3:**
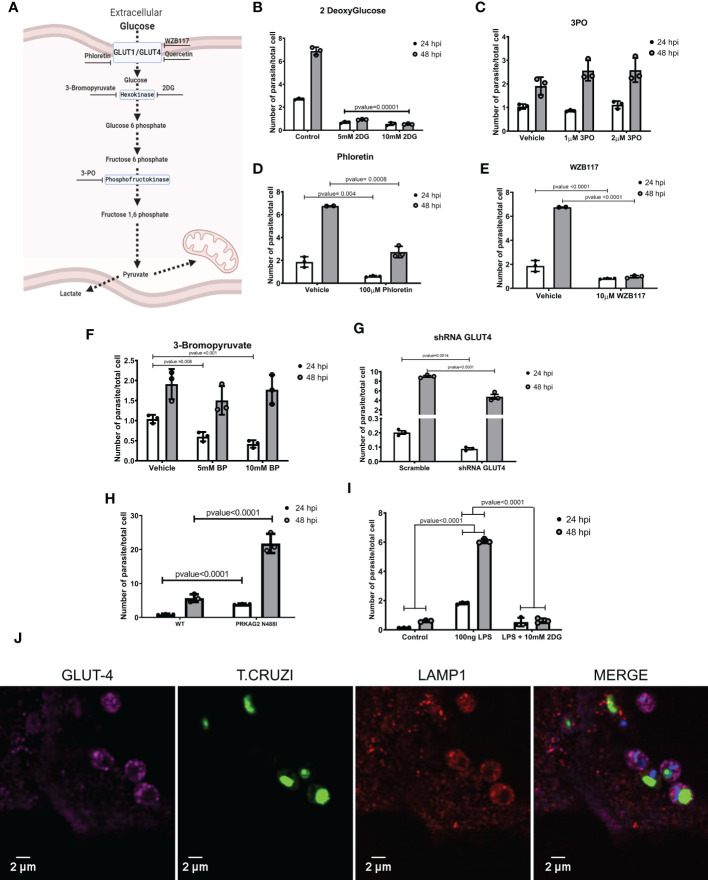
Inhibiting glycolysis impaired *T. cruzi* proliferation. **(A)** A schematic of glycolytic enzymes and inhibitors studied. **(B)** 2DG-treatment of iPSC-CMs concurrent with *T. cruzi* exposure decreased infection and cell division. **(C)** Inhibition of phosphofructokinase did not alter infection or parasite division. Inhibition of glucose transporters with **(D)** phloretin (nonspecific inhibitor) and **(E)** WZ117 (specific for GLUT1 and GLUT4) decreased infection and cell division. **(F)** Inhibition of hexokinase II by 3-bromopyruvate decreased intracellular parasites at 24 hpi but not at 48 hpi. **(G)** iPSC-CMs transduced with shRNAs targeting GLUT4 had significantly fewer intracellular parasites at 48 hpi. **(H)** PRKAG2-mutant iPSC-CMs with 2-fold higher GLUT4 levels show increased infection and parasite replication rates. **(I)** LPS-primed iPSC-CMs increased intracellular parasites at 24 hpi, while inhibition of glycolysis normalized infection rate. **(J)** Confocal images of fluorescently labeled, *T. cruzi* -infected iPSC-CMs (3 hpi) demonstrate co-localization of GLUT4 (magenta), lysosomal membrane protein 1 (red), and *T. cruzi* (green). Nuclei are DAPI stained. Data reflect three independent replicates for each experiment. Statistical analyses used ANOVA test with Bonferroni correction and p-value<0.05 was considered significant.

To further probe the effects of these agents on metabolism, we considered whether perturbations in cellular glucose and glycolysis influenced susceptibility to *T. cruzi* infection (**Figure 3A**). Pre-treatment of *T. cruzi* cultures with 2DG for 16 hours did not alter the parasite’s infectivity or replication rates (**Figure S8A**). However, iPSC-CMs treated with 2DG had 60% lower infection rates and no amastigote replication (**Figure 3B** and **Figures S8A, S8G**). Timed delivery of 2DG throughout the parasites’ life cycle (**Figures S8B, C**) demonstrated that administration within the first 4 hours after inoculation resulted in greatest attenuation of intracellular *T. cruzi* at 24 and 48 hpi. Parallel experiments in rat neonate cardiomyocytes confirmed these findings (**Figure S8D**).

As 2DG reduces intracellular glucose, we considered if 2DG-attenuated infectivity might result from de-glycosylation of membrane proteins that participate in parasite entry. To address this, we provided supplementary mannose to reduce the incorporation of 2DG in N-linked glycosylation reactions (Kurtoglu et al., 2007). Mannose did not alter the 2DG effects on parasite infection or intracellular replication (**Figure S8E**).

Pre-treatment of iPSC-CMs with 3PO, phosphofructokinase inhibitor, had no effect on parasite infection or replication (**Figure 3C**).

Because a glycolysis activation by cardiomyocytes increases glucose uptake through glucose-facilitated transporters GLUT1 and GLUT4 (Kraegen et al., 1993), we considered whether these membrane proteins might facilitate *T. cruzi* entry. As the levels of GLUT1 were indetectable in iPSC-CMs (**Figure S9B**), we only modulated GLUT4 levels. We initially tested compounds that inhibit several glucose-facilitated transporters. In comparison to vehicle-treated cells, phloretin, broadly GLUT inhibitor, significantly reduced infection and parasite replication (**Figure 3D**). Quercetin, another broadly GLUT inhibitor, silenced parasite infection and replication (**Figure S8F**), and WZB117, specific *GLUT1* and *GLUT4* inhibitor, (**Figure 3E**) reduced infection by approximately 50%.

We then employed genetic approaches to modulate *GLUT4* expression. Cells transfected with shRNAs attenuated human *GLUT4* transcripts to approximately 25% normal levels (**Figure S9A**), without causing morphologic or contractile deficits (**Figure S9C**). *GLUT4* shRNA-treated iPSCs-CM had approximately 50% lower infectivity at 24 and 48 hpi (**Figure 3G**). As these data inferred the possible involvement of *GLUT4* in *T. cruzi* infectivity, we capitalized on iPSC-CMs with an endogenous activating missense variant in *PRKAG2* that causes a 2-fold increase in *GLUT4* expression (Hinson et al., 2016). *PRKAG2*-mutant iPSC-CMs exposed to *T. cruzi* showed significantly increased infection and replication rates (**Figure 3H**).

With pharmacological and genetic data that implicated the GLUT4 transporter in parasite infection and replication, we considered if *T. cruzi* hijacked this membrane protein for cell entry. Using immunofluorescence and confocal imaging we found colocalized GLUT4, parasite, and lysosome-membrane associated protein-1 (LAMP1) in iPSC-CMs at 3 hpi (**Figure 3J**). Together these data and pharmacological studies suggest that diversion of host metabolism promoted parasite entry concurrent with activation of glycolytic metabolism.

**Figure 4:**
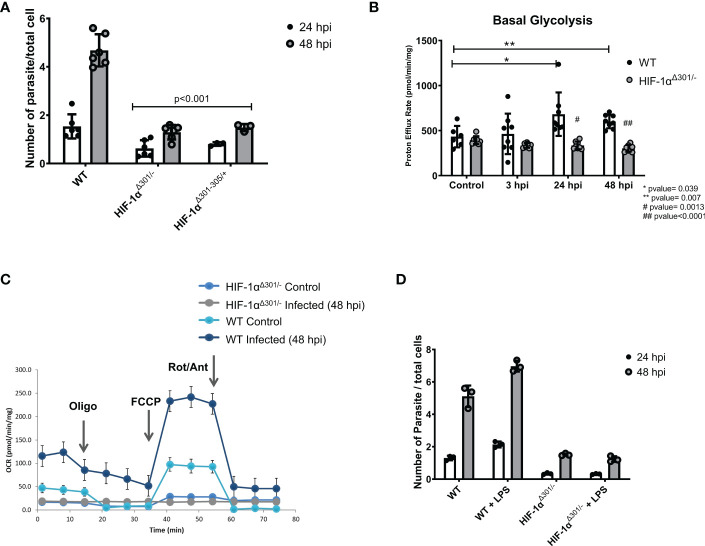
HIF-1α is required for glycolytic switch after *T. cruzi* infection. **(A)** Two iPSC-CMs lines carrying HIF-1α^Δ301/-^(compound inframe deletion of amino acid 301 and a null allele) or HIF-1α^Δ301-305/+^ (a heterozygous inframe deletion of amino acids 301- 305) have lower infection and replicate rates compared to WT cells. **(B)** HIF-1α^Δ301/-^ iPSC-CMs did not activate proton efflux, an indicator of glycolytic activation. **(C)** Extracellular metabolic flux analyses demonstrated lower mitochondrial respiratory activity (oxygen consumption rate; OCR) in HIF-1α^Δ301/-^ iPSC-CMs at 48 hpi compared to WT. **(D)** HIF-1α^Δ301/-^ iPSC-CMs were unresponsive to LPS-priming. Data reflects six **(A, B)** and three **(C, D)** independent replicates for each experiment. Statistical analyses used ANOVA test with Bonferroni correction and p-value<0.05 was considered significant.

Prior studies suggest central roles of HIF-1α signaling in both glycolytic metabolism and innate signaling in immune cells (Corcoran and O’Neill, 2016). To consider if HIF-1α similarly serves as linchpin in infected iPSC-CMs, we analyzed two independent HIF-1α mutant lines (**Figures S9D–G**). HIF-1α^Δ301-305/+^ iPSC-CMs express a heterozygous inframe deletion of five amino acids 301-305; HIF-1α^Δ301/-^ iPSC-CMs express an inframe deletion of residue 301 opposite to a null allele. In comparison to WT iPSC-CMs, HIF-1α mutant lines had 60% lowered *T. cruzi* infection rates (**Figure 4A**). Using proton efflux rates as an index of glycolysis, we found substantially reduced rewiring of metabolism in infected HIF-1α^Δ301/-^ iPSC-CMs. While proton efflux was comparable in uninfected mutant and WT iPSC-CMs (**Figure 4B**), infected HIF-1α^Δ301/-^ iPSC-CMs did not augment proton efflux nor increase oxygen consumption rates, which occurred in WT iPSC-CMs (**Figure 4C**). We interpret these data to indicate that HIF-1α activation is necessary for glycolytic activation after *T. cruzi* infection of iPSC-CMs and promotes intracellular entry and parasite replication.

### 3.4 Modulation of adaptive human cellular response suggests co-evolution of *T. cruzi* to exploit defense mechanisms

Innate immune activation with concurrent metabolic remodeling is a highly conserved response that is carried out by immune cells in response to many pathogens to benefit the host. Immune cells that are primed to activate immuno-metabolic responses are able to reduce infection rates and improve survival when subsequently inoculated with a pathogen (Cheng et al., 2014). To discern whether immuno-metabolic changes observed in infected iPSC-CMs benefited the host or parasite we used two approaches.

Our study protocol infected approximately 20% of exposed iPSC-CMs. This allowed us to consider if uninfected bystander iPSC-CMs were influenced by *T. cruzi*-mediated responses in infected cells, such as increased expression of secreted cytokines like IL-1β and IL-6. Capitalizing on the GFP-tagged parasites, we FACS-sorted infected and bystander iPSC-CMs at 48 hpi and compared RNA-seq data (**Tables S8, S9**). Remarkably, despite the considerable transcriptional changes in infected cells, RNA-seq showed no evidence for innate/immune responses or the mTOR-HIF-1α pathway activation in bystander iPSC-CMs. Additionally, bystander cells showed minimal changes in other transcripts that might suggest activation of a protective pathway.

Next, we asked whether pretreatment of iPSC-CMs with lipopolysaccharides (LPS), an endotoxin that increases innate immune-metabolic signaling and ROS production would influence *T. cruzi* infection. iPSC-CMs treated with LPS for 16 hours were viable and exhibited cell structures comparable to untreated iPSC-CMs, suggesting little or no toxicity from this treatment. In comparison to naive iPSC-CMs, pre-treatment with LPS increased *T. cruzi* infection and replication (**Figure 3J**). LPS appeared to activate the glycolysis pathway, as co-administration of 2DG with *T. cruzi* to LPS-treated iPSC-CMs normalized infection rates to that observed in naive iPSC-CMs (**Figure 3I**). Moreover, HIF-1α mutant iPSC-CMs were unresponsive to LPS pre-activation infectivity (**Figure 4D**). Comparison of RNA-seq data from LPS-treated WT and HIF-1α^Δ301/-^ iPSC-CMs showed that mutant iPSC-CMs failed to upregulate pathways involved in mTORC1 signaling, oxidative phosphorylation, and glycolysis (**Tables S12–14**).

Together our data support the conclusion that *T. cruzi* infection activates intrinsic inflammatory and immune-metabolic responses in cardiomyocytes that trigger HIF-1α-mediated metabolic rewiring. The resultant activates glycolysis, increasing levels of glucose transporters that facilitate parasite infectivity.

## 4 Discussion

We found that human iPSC-CMs infected with *T. cruzi* exhibit a genetic program that is typically found in inflammatory cells. Through transcriptomic, proteomic, and metabolomic analysis of *T. cruzi*-infected iPSC-CMs, we observed the activation of innate immune responses, including TLRs, interferons, and cytokines, which triggered the upregulation of glycolysis in iPSC-CMs through the activation of a pathway involving AKT, mTOR, and HIF-1α. This increase in glycolysis, mediated by HIF-1α, led to an increase in GLUT4 in the plasma membrane, which may facilitate *T. cruzi* entry into host cells.

Our data considerably expand prior studies that demonstrate cytokine expression in *T. cruzi* -infected iPSC-CMs (Bozzi et al., 2019), and indicate robust interferon signaling, similar to observations in other infected cell lineages, and infected patients and animal models (Kierszenbaum et al., 1995; Laucella et al., 2004; Bruno et al., 2020).

Although healthy cardiomyocytes preferentially metabolize fatty acids, infected iPSC-CMs adopted glycolysis with increased glucose consumption and lactate secretion. The same glycolysis activation was identified in CD4 T-cells during both the acute and chronic phases of *T. cruzi* infected mice (Ana et al., 2021). Additionally, upregulation of glycolysis genes such as *PFKB, PDK3* and *PGAM1* have been described in the hearts of *T. cruzi* infected mice (Bruno et al., 2020).

Oxidative phosphorylation and increased OCR described here were also identified in other studies cells (Koo et al., 2018; Libisch et al., 2018; Katherine et al., 2019; Choudhuri et al., 2021). Like activated immune cells, infected iPSC-CMs showed accumulation of succinate and increased succinate dehydrogenase expression that likely contributed to HIF-1α stabilization and activation, oxidative stress, and increased the expression of pro-inflammatory molecules IL-1β and IL-6 (Tannahill et al., 2013; Mills and O’Neill, 2014; Corcoran and O’Neill, 2016; Mills and O’Neill, 2016).

Finding that cardiomyocytes, monocytes, macrophages, and dendritic cells share a cell autonomous immune-metabolic pathway underscores its importance for pathogen control. The evolution of these responses enhances defensive roles to combat a wide range of intracellular pathogens. Indeed, biochemical activation of this pathway with LPS causes trained immunity in monocytes that elicits strong protective responses when restimulated by different pathogens including viruses, bacteria, fungi (Fecher et al., 2019), mycobacterium (Gleeson et al., 2016; Ogryzko et al., 2019) and parasites (McGettrick et al., 2016). Moreover, microbes have evolved strategies to evade this pathway, including the production of indole pyruvate by the extracellular parasite *T. brucei* that promotes HIF-1α degradation and reduces IL-1β production (McGettrick et al., 2016).

However, our data suggest that *T. cruzi* has highjacked this defensive pathway to advance parasite infection and proliferation in cardiomyocytes. By pharmacological and genetic targeting to alter different arms of the immuno-metabolic response we show that the glycolysis activation in cardiomyocytes after infection benefits the parasite, not the host. Silencing the expression of glucose transporters or 2DG-inactivation of glycolysis attenuated infection. Conversely, cardiomyocytes expressing a constitutively active *PRKAG2* variant that increases glucose transporters (Hinson et al., 2016) or LPS-treatment, increased infection, and amastigote proliferation.

These studies and immunofluorescence data that colocalize glucose transporters and *T. cruzi* within lysosomes implicate key membrane proteins in parasite entry into cardiomyocytes. HIF-1α may indirectly facilitate this process as infection (and replication) was decreased in HIF-1α-mutant iPSC-CMs. Previous studies indicating that GLUT4 translocation to the membrane is dependent on HIF-1α signaling (Sakagami et al., 2014) further supports our conclusion that HIF-1α signaling facilitates infection. Moreover, the attenuated increase in parasite infection that we observed in LPS-primed HIF-1α mutant iPSC-CMs, and in iPSC-CMs treated with both LPS and 2DG suggests that LPS effects are largely mediated by HIF-1α and glycolysis effects on cardiomyocyte metabolism.

We recognize that cell-based studies cannot fully recapitulate *in vivo* responses. iPSC-CM cultures are devoid of non-cardiomyocyte cells that reside within the myocardium as well as migratory inflammatory cells that can influence cardiomyocyte responses, cardiac architecture, and function. In addition, while this model recapitulates mechanisms related to the acute infection these may differ with chronic infection. Despite these limitations, our studies of iPSC-CMs provide new insights in the pathobiology of *T. cruzi* infected hearts. That bystander cardiomyocytes showed no pro-inflammatory or cytokine responses, despite proximity to infected iPSC-CMs, suggests that the proportion of infected cardiomyocytes may influence recruitment and activation of cytotoxic immune cells to kill *T. cruzi*. Low infection levels that escape immune detection and eradication might promote a cardiac reservoir for parasites (Burgos et al., 2010; Nagajyothi et al., 2012). Yet even low parasite burden evoked profound changes in cardiomyocyte cell biology, as was evident at 24 hpi, when 20% of cells contained only one or two intracellular parasites. Concurrent intermittent, low level cardiomyocyte lysis and reinfection of nearby cells could cause progressive cardiac deterioration as well as immunosuppression-induced reactivation of infection (Burgos et al., 2010; Nagajyothi et al., 2012). Another limitation that should be pointed is that by measuring infection at 24 hpi, it is not possible to distinguish between the initial process of infection and the subsequent survival of the parasite within the host. Both of these processes are occurring at the same time, and the measurements taken at this time point will reflect the combined effects of both infection and parasite persistence.

Other mechanisms besides active parasite replication and cardiomyocyte lysis may contribute to the emergence of chronic cardiomyopathy in 30% of *T. cruzi* infected individuals (Costa et al., 2017; Bonney et al., 2019). Innate immune responses that are trained by exposure to a pathogen or molecular mimicry can be reactivated by a distinct exposure, in part through epigenetic reprogramming (Kleinnijenhuis et al., 2012; Quintin et al., 2012; Cheng et al., 2014). Reactivation of an immuno-metabolic program established in response to other triggers, might also facilitate *T. cruzi* infection. Future epigenetic studies may help to illuminate this possibility.

Additionally, we note that two key components of this program - reduced expression of contractile genes and activation of glycolysis - would impair contractility and limit ATP production. Prior studies in animal models demonstrate that *T. cruzi* infection disrupts sarcomere and cytoskeleton proteins and elicits aberrant Ca2+ transients during both the parasite replication (Adesse et al., 2010; Manque et al., 2011; Caradonna et al., 2013; Bozzi et al., 2019) and cellular burst phases (Bilate et al., 2008). Recent findings connect inflammatory signaling with the loss of sarcomere proteins by showing that iPSC-CMs exposed to interferon gamma reduce contractile force, induce myofibrillar disarray and decrease the expression of contractile apparatus proteins (Zhan et al., 2021). Downregulation of cardiomyocyte structural proteins, induced by interferon, occurs through JAK/STAT signaling, and inhibition of this pathway abrogates sarcomere disruption (Chen et al., 2021; Zhan et al., 2021). In our study, we found JAK/STAT signaling is upregulated at both 24 hpi and 48 hpi (**Tables S4, S5**), including STAT1, STAT2, STAT3, STAT5 and JAK3 upregulation (**Tables S1, S2**).

We suggest that strategies to restore normal cardiomyocyte metabolism may attenuate *T. cruzi* infectivity, improve cardiac energetics, and reduce the emergence of cardiomyopathy and heart failure from Chagas disease.

## Data availability statement

The data presented in the study are deposited in the NCBI's Gene Expression Omnibus repository and are accessible through GEO Series accession number GSE223600 (https://www.ncbi.nlm.nih.gov/geo/query/acc.cgi?acc=GSE223600). These records are scheduled to be publicly available on Jan 28, 2023.

## Author contributions

GV designed research studies, conducted experiments, acquired, and analyzed data and wrote the manuscript. JA conducted experiments, acquired, and analyzed data. KP conducted experiments and acquired data. CT designed experiments, conducted experiments, and acquired data. JG conducted experiments and acquired data. LW designed experiments, conducted experiments, and acquired data. DB conducted experiments and provided reagents. SS provided reagents. VC, JSS, and KC conducted experiments and acquired data. JK designed research studies and provided reagents. AP designed research studies, analyzed data, and wrote manuscript. JGS designed research studies, analyzed data, and review the manuscript. CS designed research studies, analyzed data, and wrote manuscript. All authors contributed to the article and approved the submitted version.
