# Supplementary material for: Cardiomyocyte infection by Trypanosoma cruzi promotes innate immune response and glycolysis activation
Source: Front Cell Infect Microbiol. 2023 Feb 6;13:1098457. doi: 10.3389/fcimb.2023.1098457 (PMC9940271; doi:10.3389/fcimb.2023.1098457)
Supplement: Supplementary file 1 [file DataSheet_1.docx]

**Detailed Methods:**

**Reagents and chemicals:**

*Cells*

LLC-MK2 culture cells (ATCC CCL-7; American Type Culture Collection, Rockville, MD)

*Cell culture media reagents*

RPMI 1640 medium (#11875-093; Gibco, Carlsbad, CA); Fetal bovine serum (FBS #16000044; Gibco, Carlsbad, CA); B27 supplemented 50x (#17504044, Gibco, Carlsbad, CA), B25 minus insulin supplemented 50x (#A1895601, Gibco, Carlsbad, CA), DMEM low glucose (#11885084, Gibco, Carlsbad, CA), 199 (#11150059, Gibco, Carlsbad, CA), Newborn Calf Serum (NBCS, #16010142, Gibco, Carlsbad, CA), Horse Serum (HRS, # 16050114, Gibco, Carlsbad, CA), Matrigel (#354234, Corning, USA), GlutaMAX (#35050061, Gibco, Carlsbad, CA), Sodium pyruvate (#11360070, Gibco, Carlsbad, CA), Glucose (#A2494001, Gibco, Carlsbad, CA), mTeSR (#85850, Stemcell Technologies, USA).

*Antibodies*

Anti-ERK1/2 (#9102, Cell Signaling, USA), Anti-Phospho ERK1/2 (#4377, Cell Signaling, USA), Anti-Phospho AKT(S473) (#4060, Cell Signaling, USA), Anti- AKT (#9272, Cell Signaling, USA), Anti-βactin (#ab8227, Abcam, USA), Anti-HIF-1α (#3716, Cell Signaling, USA), Anti-GLUT4 (#sc-53566, Santa Cruz, USA), Anti-GLUT1 (#12939S, Cell Signaling, USA), Anti-NA+/K ATPase (#ab76020, Abcam, USA).

*Kits*

BCA Protein Assay (#23227, Thermo Scientific, USA), Seahorse XF Cell Mito Stress Test Kit (#103015-100, Agilent Technologies), Seahorse XF Glycolytic Rate Assay Kit (#103344-100, Agilent Technologies), Human Stem Cell Nucleofector Kit AMAXA (#VAPH-5022, Lonza, Germany), prepGEM®Tissue Kit (#95044-032, zyGEM, UK), QIAprep Spin Miniprep Kit (#27104, Qiagen, USA), Zero Blunt TOPO PCR Cloning Kit (#K287520, Thermo Scientific, USA), SLC2A1 – GLUT1 Human shRNA Plasmid Kit (#TL309344, Origene, USA), SLC2A4 – GLUT4 Human shRNA Plasmid Kit (#TL309342, Origene, USA), Subcellular Protein Fractionation Kit for Cultured Cells (#78840, Thermo Scientific, USA)

*Chemicals*

Paraformaldehyde (PFA #158127, Sigma-Aldrich, Saint Louis, MO); Casein (#C3400, Sigma-Aldrich, Saint Louis, MO); Bovine serum albumin (BSA # A9418; Sigma-Aldrich, Saint Louis, MO); Acetonitrile (LC-MS CHROMASOLV - #34967, Sigma-Aldrich, Germany), 2-propanol (LC-MS Ultra CHROMASOLV - #34965, Sigma-Aldrich, Germany), Pyridine (LC-MS CHROMASOLV Plus - #270407, Sigma-Aldrich, USA), Methoxyamine hydrochloride (#226904, Sigma-Aldrich, USA), FAME (Fatty Acid Methyl Ester #18919- Sigma-Aldrich, USA), MSTFA (N-methyl-N-trimethylsilyltrifluoroacetamine #69479, Sigma-Aldrich, Switzerland), TMCS (Trimethylchlorosilane #89585, Sigma-Aldrich, USA), Myristic Acid D27 (#366889, Sigma-Aldrich, USA). Urea (#U5128, Sigma-Aldrich, USA); Iodoacetamide (IAA, #I6125, Sigma-Aldrich, USA); Ammonium Bicarbonate (#A6141, Sigma-Aldrich, USA); Trypsin (# V511A, Promega, USA); Trifluoroacetic Acid (TFA #40967, Sigma-Aldrich, USA); DTT (#15508-013, Thermo Scientific, USA); Collagenase type II (#234155, Sigma-Aldrich, USA), Pancreatin (#P3292, Sigma-Aldrich, USA), Duraguard capillary column (Agilent 122-5532G), DAPI (# D9532; Sigma-Aldrich, USA), PVDF membrane (#10600021, GE Life Science), C18 ZipTip (#ZTC18M960, Millipore, USA), Alexa Fluor 555 Phalloidin (#A34055, Thermo Scientific, Waltham, MA), CHIR-99021-HCL (#orb154612, Biorbyt, USA), Y-27632-HCL (#orb154624, Biorbyt, USA), Wnt-C59 (#orb181132, Biorbyt, USA), Sodium Lactate (#L7022, Sigma-Aldrich, USA), TriZOL Reagent (#15596026, Thermo Scientific, USA), Bromodeoxyuridine (#B5002, Sigma-Aldrich, USA), DMSO (#D2650, Sigma-Aldrich, USA).

**Drug treatments**

Table 1 – Drugs used to treat iPSC-CM during pathways activation or inhibition, their mechanism of action, concentration used, solvent diluted (when not media), source, identification number and reference.

| Drug (name) | Target/mechanism of action | Concentration used | Source/ID | Reference |
| --- | --- | --- | --- | --- |
| 2-Deoxyglucose | Hexokinase inhibitor. Glucose analog 2-deoxy-D-glucose (2DG) enters cells through glucose transporters, undergoes phosphorylation by hexokinase and thereby potently inhibits its physiologic activity (Ihrlund et al., 2008), and blocks the production of fructose-6 phosphate by glucose-6-isomerase. | 5 mM, 10 mM and 20 mM | Sigma Aldrich #D6134 | (Tannahill et al., 2013) |
| WZB117 | GLUT1 and GLUT 4 inhibitor (Ojelabi et al., 2016), | 10 µM (DMSO) | Sigma Aldrich #SML0621 | (Xintaropoulou et al., 2015) |
| Quercetin | Competitive inhibitor of GLUT1 (Hamilton et al., 2018), GLUT2 (Kwon et al., n.d.), GLUT3 (Park & Levine, 2000) and GLUT4 (Strobel et al., 2005) | 50 µM and 100 µM (DMSO) | Sigma Aldrich #Q4951 | (Xintaropoulou et al., 2015) |
| Phloretin | Non-specific inhibitor of sodium-glucose transporter SGLT1 and SGLT2 (Rossetti et al., 1987) and a strong GLUT1, GLUT2 and GLUT4 inhibitor (And & Ball, 1964; Cao et al., 2007; Granchi et al., 2016; Wood et al., 2008) | 100 µM (DMSO) | Sigma Aldrich #524488 | (Xintaropoulou et al., 2015) |
| 3-Bromopyruvate | Hexokinase II inhibitor | 5 mM and 10 mM (DMSO) | Sigma Aldrich #16490 | (Xintaropoulou et al., 2015) |
| 3PO (3-(3-pyridinyl)-1-(4-pyridinyl)-2-propen-1-one) | Cell-permeable, selective inhibitor of phosphofructokinase which affects the oxidative step of glycolysis (Clem et al., 2008). | 1 µM and 2 µM (DMSO) | Sigma Aldrich #525330 | (Xintaropoulou et al., 2015) |
| LPS (Lipopolysaccharides) | Pro-inflammatory endotoxin that increases innate immune-metabolic signaling and ROS production (Cascante et al., 2010; Koo et al., 2016; Mills et al., 2016; Mills & O’Neill, 2016; Zhang et al., 2010). | 100 ng/mL | Sigma Aldrich #L5293 | (Tannahill et al., 2013) |
| Resveratrol | ROS scavenger. Activates AMPK (5' AMP-activated protein kinase), SIRT1 (sirtuin-1), and PCG-1α (peroxisome proliferator-activated receptor-gamma coactivator-1α) and reduces ROS by stabilizing the mitochondrial membrane potential (Baur & Sinclair, 2006). | 0 μM, 20 μM and 50 μM (DMSO) | Sigma Aldrich #R5010 | (Valera Vera et al., 2016) |

**LC-MS**

The samples were processed by a nanoACQUITY (Waters) system comprising a binary pump, an auxiliary pump, and a sampler. Peptides were captured, desalted, and concentrated in a capture column Symmetry C18 (20 mm ×180 mm, 5 µm) using a mobile phase composed of water with 0.1% TFA at a flow rate of 15 µL/min for 5 minutes. Then, peptides were separated on an analytical column HSSC18 (75 μm × 150 mm, 1.7 μm) by eluting with a linear gradient of 2% DMSO in water with 0.1% formic acid and 5% DMSO in acetonitrile with 0.1% formic acid. The proportion of the organic solution was increased from 0 to 60% in 80 minutes.

The chromatographic system is directly coupled to a hybrid quadrupole orbitrap tandem mass spectrometer Q-Exactive (Thermo Scientific), equipped with a Nano Flex source. The acquisition of spectral data was obtained by the data dependent top-15 method in which the spectrometer chooses dynamically the most abundant not-yet sequenced precursor ions from a survey scan from 390 to 1650 m/z (except for the monocharged and those with charges exceeding 7) at 70,000 (at m/z 200) of resolution and AGC target 5e6. Sequencing was achieved dissociating the precursor ion with normalized collision energy of 35, resolution equal to 17,500 and AGC target of 5e4.

Spectra were processed using Maxquant version 1.5.1.2 for peptide and protein identification as described (Cox & Mann, 2008) using the SwissProt Proteome Human reference (Human 9606) and *T. cruzi* proteome reference (*T. cruzi* 5693) from Uniprot. MS/MS database search was performed using default settings, with a 10ppm mass tolerance for the main search. Cysteine carbamidomethylation was selected as a fixed modification and acetyl NH_2_-terminal and oxidation (methionine) were selected as variable modifications. Trypsin was selected as the protease, with up to two missed cleavages allowed. Results were filtered by a 0.01 false discovery rate at both protein and peptide levels. The minimum length of acceptable identified peptides was set as seven amino acids. The contaminants were eliminated based on a library of contaminants from MaxQuant. Proteins with p-value different from 1 were accepted. Label-free quantification was obtained through normalized (LFQ) data in MaxQuant.

**GC-MS**

One microliter of each sample was injected into an Agilent 7890B GC system operated in splitless mode. The metabolites were separated in a DB5-MS + 10m Duraguard capillary column with helium carrier gas flowed at a rate of 1.1mL min -1. The injector temperature was set at 250ºC. The column temperature was held at 60ºC for 1 min, and then increased to 310ºC at a rate of 10ºC/min for 37 minutes. The column effluent was introduced into the ion source of an Agilent 5977A mass selective detector. The detector operated in the electron impact ionization mode (70 eV), mass spectra were acquired in scan mode with range between 50 and 500m/z and recorded after a solvent delay of 6.5 min with 3 scans per second. The MS quadrupole temperature was set at 180ºC and the ion source temperature was set at 280ºC. Each sample was analyzed in three technical replicates.

Identification of compounds was made comparing the mass spectra and retention time (RT) of all detected compounds with the Agilent Fiehn GC/MS Metabolomics RTL Library (version A.02.02) and the National Institute of Standards and Technology (NIST) library 11 (2014) using Unknowns–- Agilent MassHunter Workstation Quantitative Analysis (version B.06.00).

**Western blot experiments**

iPSC-CM baseline and after infection (1x10^6^ cells) were washed with PBS and harvest with 300μL of protein extraction buffer (8 M urea and 50 mM of ammonium bicarbonate). Proteins were measured with BCA methods according to manufacturer. Proteins were loaded into 10% SDS-PAGE and transferred to a PVDF membrane in a wet system. Membranes were blocked with 5% BSA diluted in PBS and incubated with antibodies against HIF-1α, phospho-AKT, total AKT, phospho-ERK, total ERK, GLUT4 and β-actin overnight at 4°C. Cell membrane was extracted using Subcellular Protein Fractionation Kit for Cultured Cells (Thermo) according to manufacturer recommendation. Membranes were incubated with HRP secondary antibodies and visualized in GE ImageQuant LAS 4000. Bands were measured using ImageJ.

**Flow Cytometry Sorting**

Forty-eight (48) hours after infection, iPSC-CMs were trypsinized and single cells were stained with propidium iodide for 30 minutes. Cells were washed and sorted using MoFlo Astrios Cell Sorter (Beckman Coulter). Propidium iodide-stained cells were discarded and non- propidium iodide-stained cells were sorted in GFP-positive and GFP negative. Cells were kept in ice and fresh PBS until RNA extraction.

**Primary culture of Rat cardiomyocytes**

Rat (*Rattus norvegicus*, Wistar) neonatal cardiomyocyte extraction was performed as described (Jensen et al., 2018). Briefly, animals were decapitated 24–48 hours after birth. Heart ventricles were removed, diced into 1-3mm pieces, and washed with ADS buffer (EdgeBio). After washing, pieces were transferred to a 50 mL Falcon tube with 3 mL of collagenase type II and pancreatin (0.2 mg/ml) for digestion and then incubated at 37°C shaking (340xg) for 10 minutes. The supernatant was removed and added NBCS for enzyme blocking. Cells were centrifuged at 340g for 5 minutes. The supernatant was carefully poured off and the remaining pellet was suspended with 10 mL media and retained at 37°C and 5% CO2 incubator. This digestion procedure was repeated six times. At the end of digestion, cells were flowed through 100 µm cell strainer followed by a washing step with fresh medium into an empty Falcon. The content was plated in a T150 flask during 45 minutes at 37°C for fibroblasts adhesion. The supernatant was centrifuged at 340xg for 5 minutes; then the pellet re-suspended in the 15 mL of media containing DMEM-Low glucose and 199 mixtures (4:1) with 10% of HRS (Horse Serum), 5% of NBCS, and 1% of Bromodeoxyuridine (BrdU). Cells were counted and plated on laminin coated plates in 105 cells/cm^2^.

**Nitric oxide detection assay.**

The release of nitric oxide (NO) from iPSC-CM after infection was measured as nitrite (NO_−2_) accumulation in the culture media as previously described (Venturini et al., 2019). We analyzed the chemiluminescence reaction between ozone and the NO generated by reduction of the sample with vanadium chloride in acid at 95°C using NO analyzer (model 208A; Sievers Instruments, Boulder, CO) according to the manufacturer’s protocols. NO_−2_ levels were corrected for total protein content of iPSC-CM extracts and media volume. The rates of NO_−2_ accumulation are expressed as micromoles per grams of protein per milliliters of medium. Three samples were analyzed.

**References:**

And, F., & Ball, E. G. (1964). Studies on the Metabolism of Adipose Tissue. XVI. Inhibition by Phlorizin and Phloretin of the Insulin-stimulated Uptake of Glucose*. In *ACTION OF PHLORIZIN AND PHLORETIN ON ADIPOSE TISSUE* (Vol. 3, Issue 7).

Baur, J. A., & Sinclair, D. A. (2006). Therapeutic potential of resveratrol: The in vivo evidence. In *Nature Reviews Drug Discovery*. https://doi.org/10.1038/nrd2060

Cao, X., Fang, L., Gibbs, S., Huang, Y., Dai, Z., Wen, P., Zheng, X., Sadee, W., & Sun, D. (2007). Glucose uptake inhibitor sensitizes cancer cells to daunorubicin and overcomes drug resistance in hypoxia. *Cancer Chemotherapy and Pharmacology*, *59*(4), 495–505. https://doi.org/10.1007/s00280-006-0291-9

Cascante, M., Bosca, L., Traves, P. G., Rodriguez-Prados, J.-C., Martin-Sanz, P., Cuenca, J., Rico, D., & Aragones, J. (2010). Substrate Fate in Activated Macrophages: A Comparison between Innate, Classic, and Alternative Activation. *The Journal of Immunology*. https://doi.org/10.4049/jimmunol.0901698

Clem, B., Telang, S., Clem, A., Yalcin, A., Meier, J., Simmons, A., Rasku, M. A., Arumugam, S., Dean, W. L., Eaton, J., Lane, A., Trent, J. O., & Chesney, J. (2008). Small-molecule inhibition of 6-phosphofructo-2-kinase activity suppresses glycolytic flux and tumor growth. *Molecular Cancer Therapeutics*, *7*(1), 110–120. https://doi.org/10.1158/1535-7163.MCT-07-0482

Granchi, C., Fortunato, S., & Minutolo, F. (2016). Anticancer agents interacting with membrane glucose transporters. In *MedChemComm* (Vol. 7, Issue 9, pp. 1716–1729). Royal Society of Chemistry. https://doi.org/10.1039/c6md00287k

Hamilton, K. E., Rekman, J. F., Gunnink, L. K., Busscher, B. M., Scott, J. L., Tidball, A. M., Stehouwer, N. R., Johnecheck, G. N., Looyenga, B. D., & Louters, L. L. (2018). Quercetin inhibits glucose transport by binding to an exofacial site on GLUT1. *Biochimie*, *151*, 107–114. https://doi.org/10.1016/j.biochi.2018.05.012

Ihrlund, L. S., Hernlund, E., Khan, O., & Shoshan, M. C. (2008). 3-Bromopyruvate as inhibitor of tumour cell energy metabolism and chemopotentiator of platinum drugs. *Molecular Oncology*, *2*(1), 94–101. https://doi.org/10.1016/j.molonc.2008.01.003

Jensen, L., Neri, E., Bassaneze, V., de Almeida Oliveira, N. C., Dariolli, R., Turaça, L. T., Levy, D., Veronez, D., Ferraz, M. S. A., Alencar, A. M., Bydlowski, S. P., Cestari, I. A., & Krieger, J. E. (2018). Integrated molecular, biochemical, and physiological assessment unravels key extraction method mediated influences on rat neonatal cardiomyocytes. *Journal of Cellular Physiology*. https://doi.org/10.1002/jcp.26380

Koo, S. J., Chowdhury, I. H., Szczesny, B., Wan, X., & Garg, N. J. (2016). Macrophages promote oxidative metabolism to drive nitric oxide generation in response to Trypanosoma cruzi. *Infection and Immunity*, *84*(12), 3527–3541. https://doi.org/10.1128/IAI.00809-16

Kwon, O., Eck, P., Chen, S., Corpe, C. P., Lee, J.-H., Kruhlak, M., & Levine, M. (n.d.). Inhibition of the intestinal glucose transporter GLUT2 by flavonoids. *The FASEB Journal • Research Communication*. https://doi.org/10.1096/fj.06-6620com

Mills, E. L., Kelly, B., Logan, A., Costa, A. S. H., Varma, M., Bryant, C. E., Tourlomousis, P., Däbritz, J. H. M., Gottlieb, E., Latorre, I., Corr, S. C., McManus, G., Ryan, D., Jacobs, H. T., Szibor, M., Xavier, R. J., Braun, T., Frezza, C., Murphy, M. P., & O’Neill, L. A. (2016). Succinate Dehydrogenase Supports Metabolic Repurposing of Mitochondria to Drive Inflammatory Macrophages. *Cell*. https://doi.org/10.1016/j.cell.2016.08.064

Mills, E. L., & O’Neill, L. A. (2016). Reprogramming mitochondrial metabolism in macrophages as an anti-inflammatory signal. *European Journal of Immunology*. https://doi.org/10.1002/eji.201445427

Ojelabi, O. A., Lloyd, K. P., Simon, A. H., De Zutter, J. K., & Carruthers, A. (2016). WZB117 (2-fluoro-6-(m-hydroxybenzoyloxy) Phenyl m-Hydroxybenzoate) inhibits GLUT1-mediated sugar transport by binding reversibly at the exofacial sugar binding site. *Journal of Biological Chemistry*, *291*(52), 26762–26772. https://doi.org/10.1074/jbc.M116.759175

Park, J. B., & Levine, M. (2000). Intracellular accumulation of ascorbic acid is inhibited by flavonoids via blocking of dehydroascorbic acid and ascorbic acid uptakes in HL-60, U937 and Jurkat cells. *Journal of Nutrition*, *130*(5), 1297–1302. https://doi.org/10.1093/jn/130.5.1297

Rossetti, L., Smith, D., Shulman, G. I., Papachristou, D., & DeFronzo, R. A. (1987). Correction of hyperglycemia with phlorizin normalizes tissues sensitivity to insulin in diabetic rats. *Journal of Clinical Investigation*, *79*(5), 1510–1515. https://doi.org/10.1172/JCI112981

Strobel, P., Allard, C., Perez-Acle, T., Calderon, R., Aldunate, R., & Leighton, F. (2005). Myricetin, quercetin and catechin-gallate inhibit glucose uptake in isolated rat adipocytes. *Biochemical Journal*, *386*(3), 471–478. https://doi.org/10.1042/BJ20040703

Tannahill, G. M., Curtis, A. M., Adamik, J., Palsson-McDermott, E. M., McGettrick, A. F., Goel, G., Frezza, C., Bernard, N. J., Kelly, B., Foley, N. H., Zheng, L., Gardet, A., Tong, Z., Jany, S. S., Corr, S. C., Haneklaus, M., Caffrey, B. E., Pierce, K., Walmsley, S., … O’Neill, L. A. J. (2013). Succinate is an inflammatory signal that induces IL-1β through HIF-1α. *Nature*, *496*(7444), 238–242. https://doi.org/10.1038/nature11986

Valera Vera, E. A., Sayé, M., Reigada, C., Damasceno, F. S., Silber, A. M., Miranda, M. R., & Pereira, C. A. (2016). Resveratrol inhibits Trypanosoma cruzi arginine kinase and exerts a trypanocidal activity. *International Journal of Biological Macromolecules*, *87*, 498–503. https://doi.org/10.1016/j.ijbiomac.2016.03.014

Venturini, G., Malagrino, P. A., Padilha, K., Tanaka, L. Y., Laurindo, F. R., Dariolli, R., Carvalho, V. M., Cardozo, K. H. M., Krieger, J. E., & Pereira, A. C. (2019). Integrated proteomics and metabolomics analysis reveals differential lipid metabolism in human umbilical vein endothelial cells under high and low shear stress. *American Journal of Physiology - Cell Physiology*, *317*(2). https://doi.org/10.1152/ajpcell.00128.2018

Wood, T. E., Dalili, S., Simpson, C. D., Hurren, R., Mao, X., Saiz, F. S., Gronda, M., Eberhard, Y., Minden, M. D., Bilan, P. J., Klip, A., Batey, R. A., & Schimmer, A. D. (2008). A novel inhibitor of glucose uptake sensitizes cells to FAS-induced cell death. *Molecular Cancer Therapeutics*, *7*(11), 3546–3555. https://doi.org/10.1158/1535-7163.MCT-08-0569

Xintaropoulou, C., Ward, C., Wise, A., Marston, H., Turnbull, A., & Langdon, S. P. (2015). A comparative analysis of inhibitors of the glycolysis pathway in breast and ovarian cancer cell line models. *Oncotarget*, *6*(28), 25677–25695. https://doi.org/10.18632/oncotarget.4499

Zhang, S., Kim, C. C., Batra, S., McKerrow, J. H., & Loke, P. (2010). Delineation of Diverse Macrophage Activation Programs in Response to Intracellular Parasites and Cytokines. *PLoS Neglected Tropical Diseases*, *4*(3), e648. https://doi.org/10.1371/JOURNAL.PNTD.0000648

**Supplementary Figures**

**S1 Fig**

**
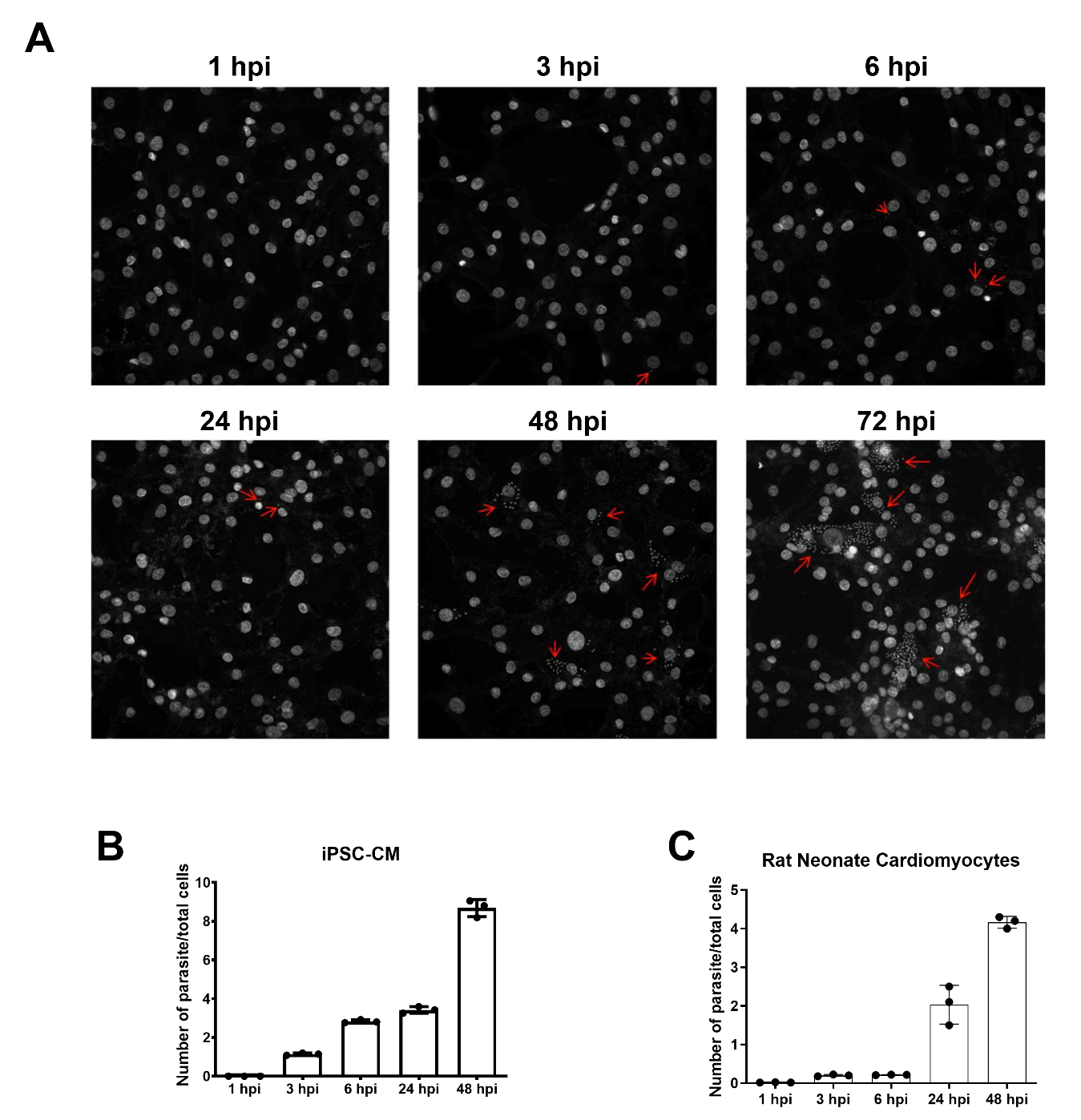
**

**S1 Fig**: Time course of *T. cruzi* infection in iPSC-CMs. With a MOI of 5:1 (parasite:cell), 20% of iPSC-CMs *T. cruzi* become infected. Intracellular parasites are present at 3 hpi and infection increases through 6 hpi. The numbers of intracellulalar parasites did not increase between 6 and 24 hpi, but did so during replication phase from 24 to 48 hpi (**B)**. The same time-couse was observed for both iPSC-CMs and rat neonate cardiomyocytes (**C**). Data reflects analyses of three independent replicate samples for each experiment. (**A)**. Host and *T. cruzi* nuclei in gray. Parasites are indicated with red arrow.

**S2 Fig**

**
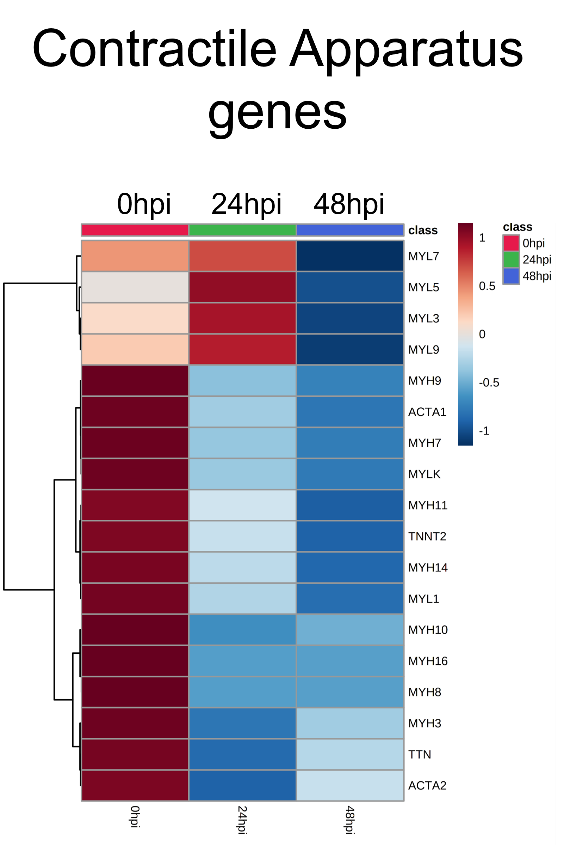
**

**S2 Fig.** Heatmap of differentially downregulated genes involved in contractile apparatus.

**S3 Fig**

**
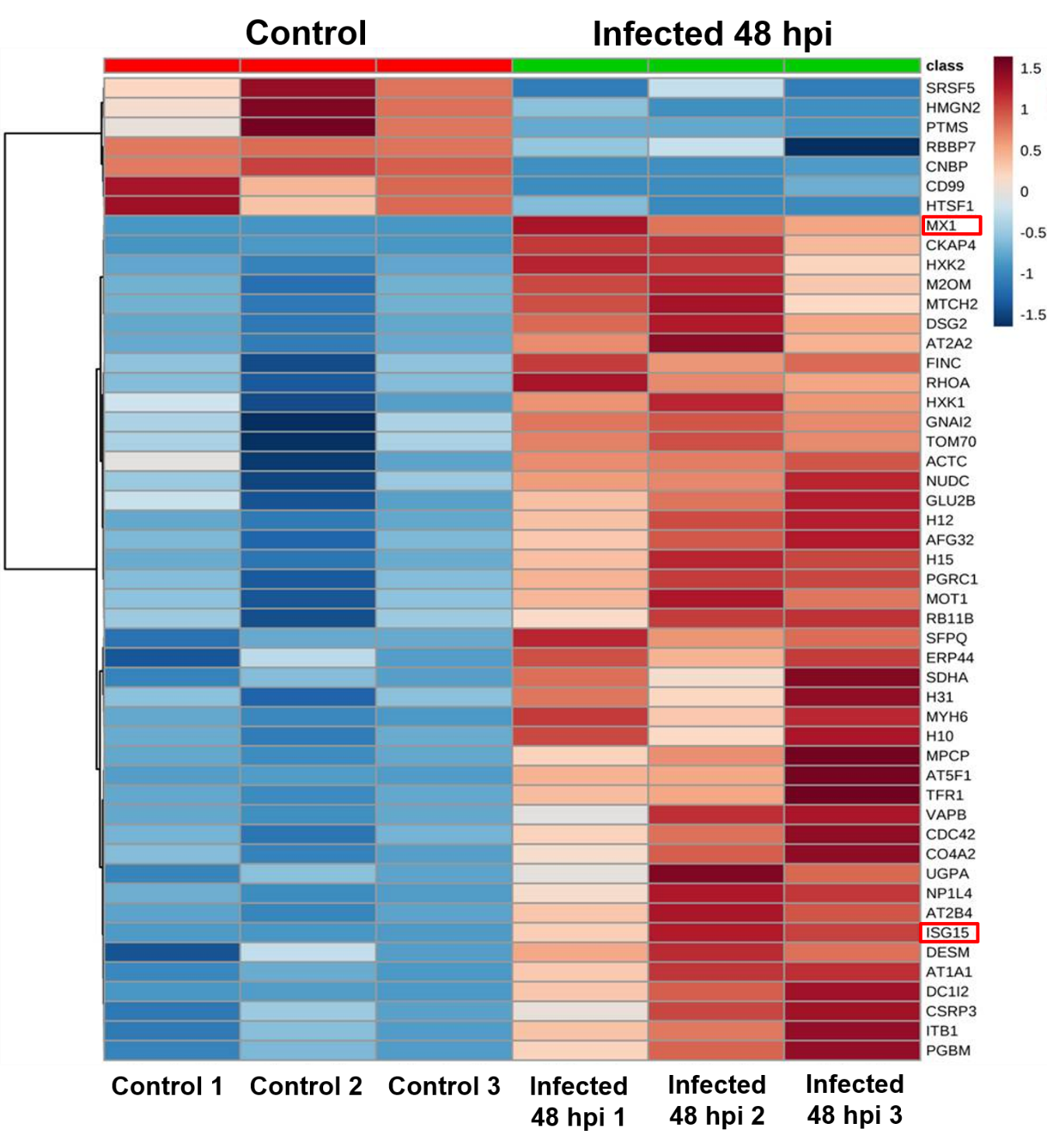
**

**S3 Fig.** The proteomic signature of *T. cruzi*-infected iPSC-CMs. The heat map representation overall shotgun proteomic analyses and highlights (red box) interferon, glycolysis and hypoxia signaling proteins. Data reflects analyses of three independent replicate samples for each experiment. Statistical analyses used T-test and p-value<0.05 was considered significant.

**S4 Fig**

**
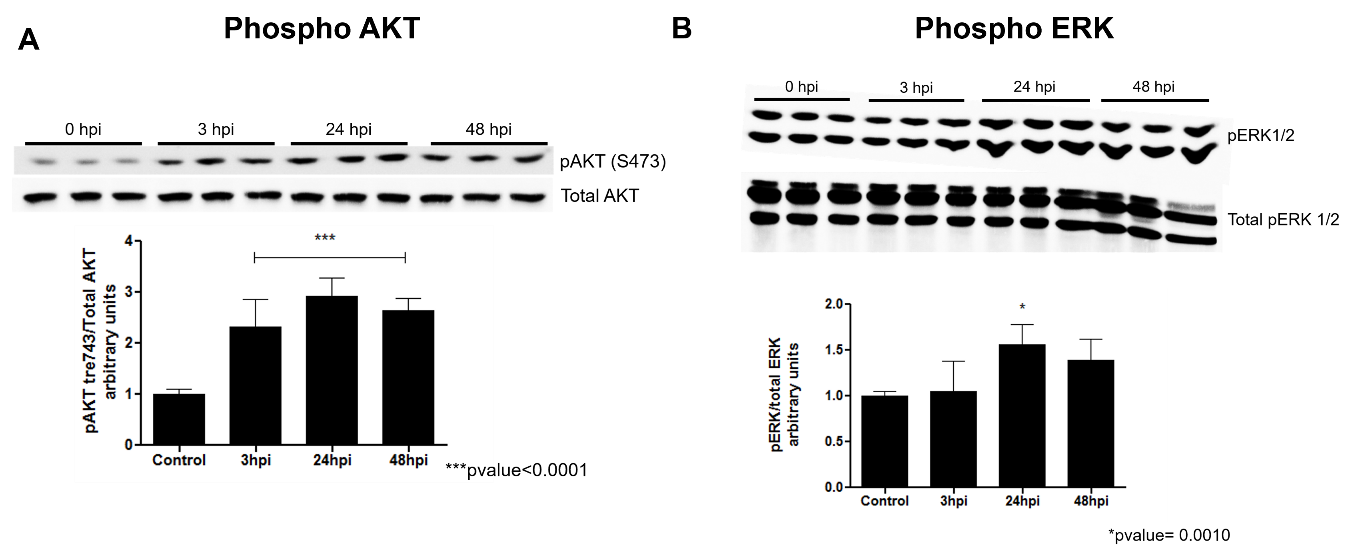
**

**S4 Fig.** *T. cruzi* infection increased HIF-1α expression and activated HIF-1α pathway in iPSC-CMs, including (**A)** AKT-phosphorylation and (**B)** ERK-phosphorylation. Data shows results from three independent replicates for each experiment. Statistical analyses used ANOVA test with Bonferroni correction and p-value<0.05 was considered significant.

**S5 Fig**

**
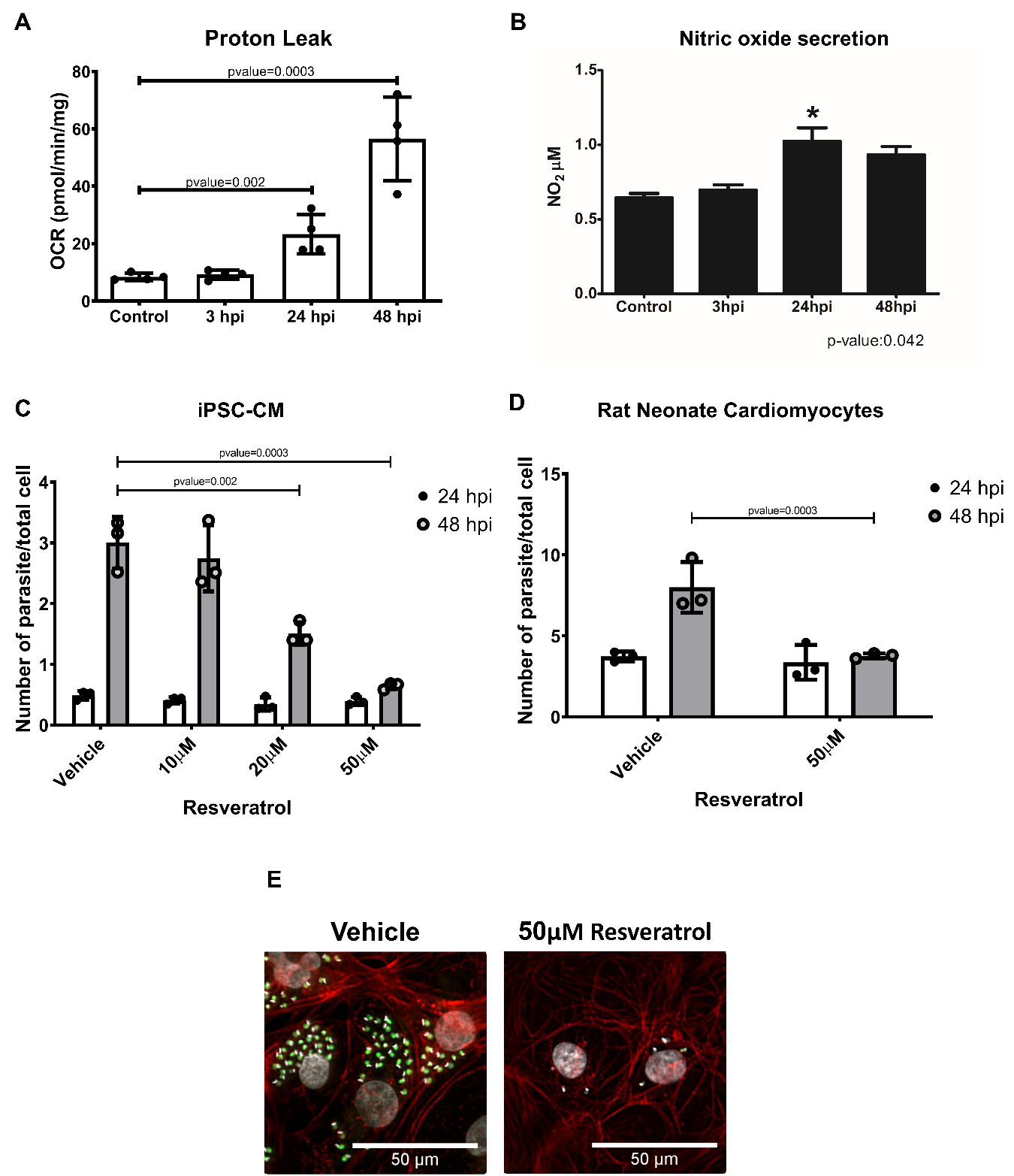
**

**S5 Fig.** Oxidative stress is increased in *T. cruzi*- infected iPSC-CMs. **A**. Proton leak, measured by extracellular metabolic flux analyses, is increased in iPSC-CMs at 24 hpi and 48 hpi. **B.** Nitric oxide (measured as nitrite) in the media after parasite infection. **C.** Resveratrol, a non-specific antioxidant, decreased *T. cruzi* replication rate at 48 hpi in iPSC-CMs (**C**) and in rat neonatal cardiomyocytes (**D**). **E** Fluorescence images of *T. cruzi*- infected iPSC-CMs with and without Resveratrol treatment. GFP-tagged *T. cruzi*, green; host and parasite DAPI-stained nuclei, grey; alexa-555 phalloidin-stained actin cytoskeleton, red. Data reflects analyses of three independent replicate samples for each experiment. Statistical analyses used ANOVA test with Bonferroni correction and p-value<0.05 was considered significant.

**S6 Fig**

**
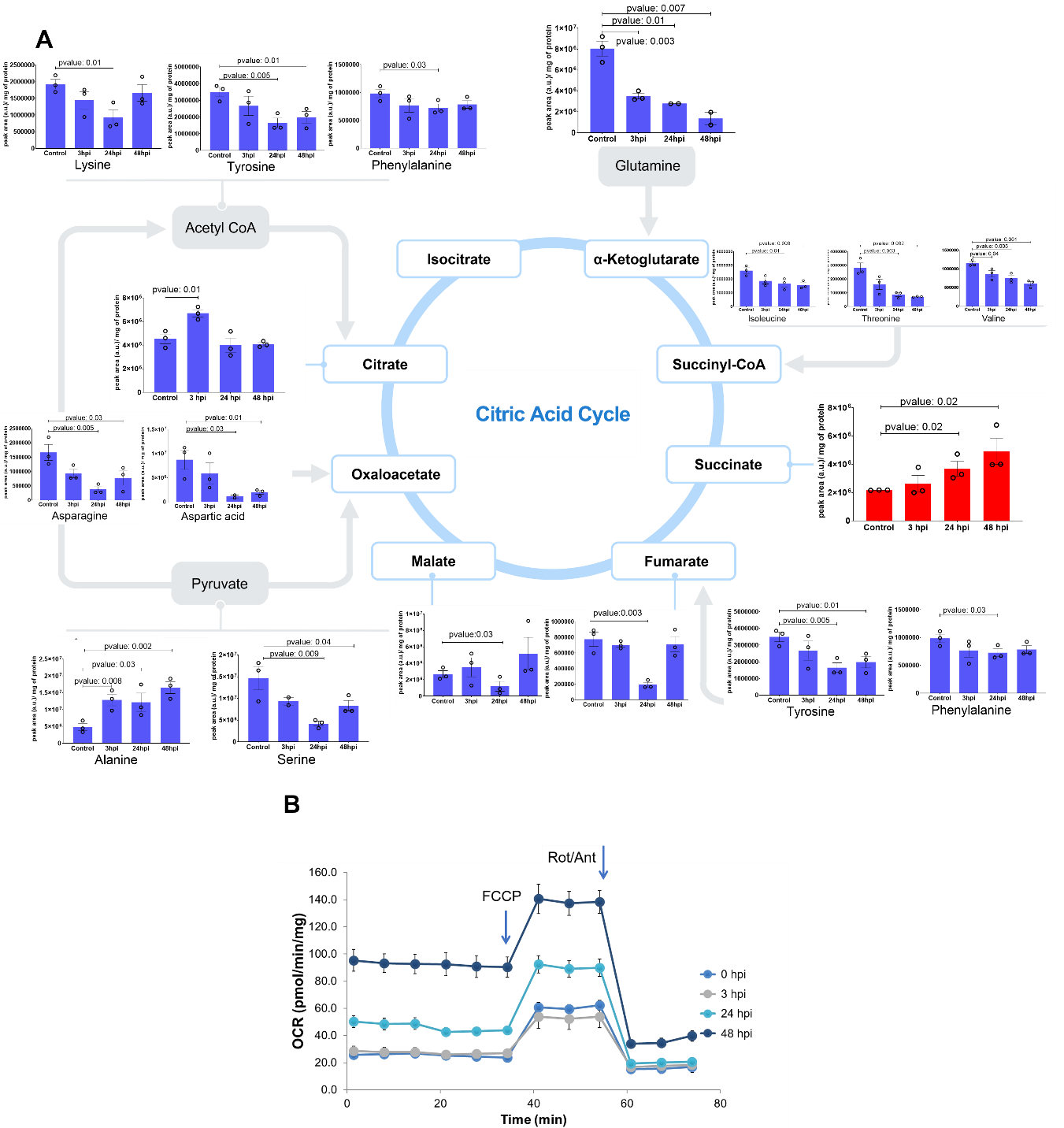
**

Oligo

**S6 Fig.** Metabolic changes in iPSC-CMs accompany *T. cruzi* infection. **A.** Changes in TCA metabolites included (i) an early increase in citrate (3 hpi), (ii) progressive increases in succinate (red) over the infection time, (iii) reduced levels of fumarate and malate at 24 hpi, followed by increased malate levels at 48 hpi. Amino acid catabolism that are likely to contribute to altered metabolites are shown and suggest anaplerotic processes contribute to TCA intermediates, as occur in inflammatory cells. Data reflects analyses of three independent replicate samples for each experiment. **B.** Extracellular metabolic flux analyses show increased mitochondrial respiratory activity (oxygen consumption rate; OCR) at in iPSC-CMs at 24 hpi and 48 hpi. Statistical analyses used ANOVA test with Bonferroni correction and p-value<0.05 was considered significant.

**S7 Fig**

**
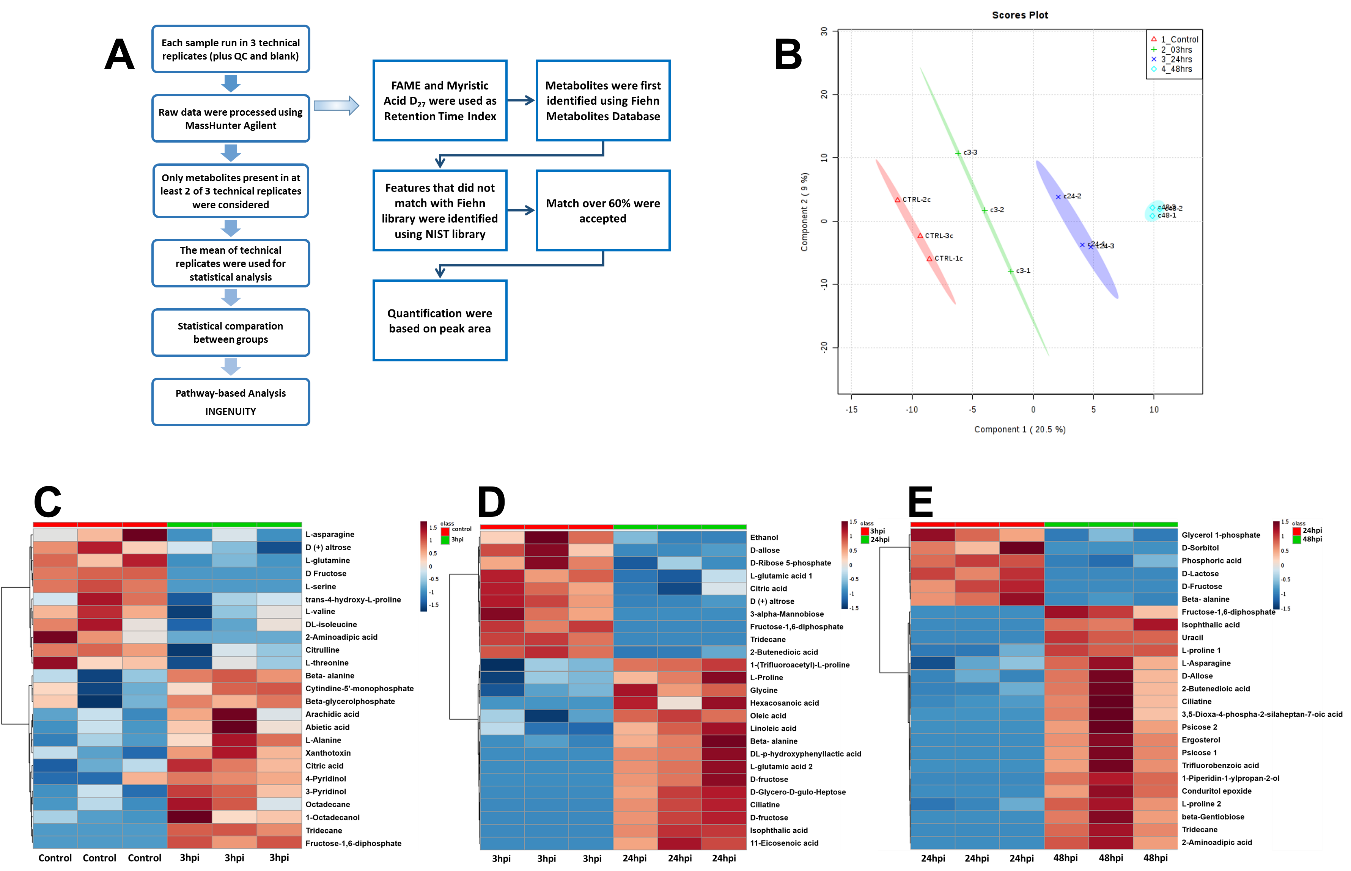
**

**S7 Fig:** Metabolomic analyses of *T. cruzi* infected iPSC-CMs. **A**. 311 metabolites were identified and quantified at baseline 3, 24, and 48 hpi. Data reflect three independent replicates per time point. **B**. Principal component analyses from all metabolites separated samples according to time post infection. **C, D**, and **E**. Metabolite shifts were assessed from heat maps derived from hierarchical clustering analyses at each time point. Metabolites with statistically different mean values are shown between baseline (0 hpi) versus 3 hpi (panel **C**), 3hpi versus 24 hpi (panel **D**), and 24 hpi versus 48 hpi (panel **E**). Comparisons at 3 hpi, 24 hpi and 48 hpi identified 29, 46, and 55 metabolites were increased or decreased, respectively. Statistical analyses used T-test p-value<0.05 was considered significant.

**S8 Fig**

**
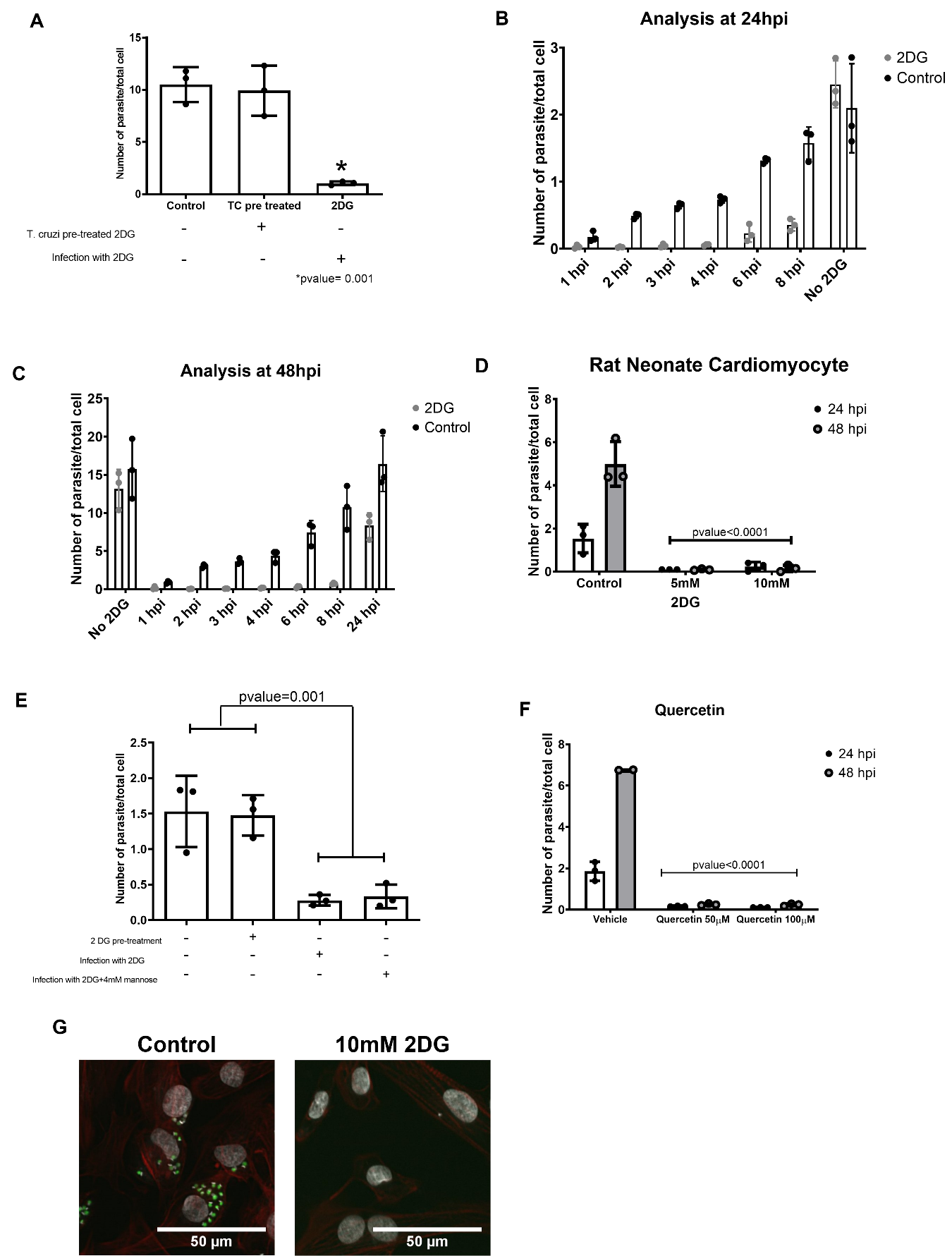
**

**S8 Fig.** Altering glycolysis impacts *T. cruzi* infection of iPSC-CMs. **A.** Pre-treatment of trypomastigotes with 10mM of 2DG for 16 hours prior to infection did not alter parasites infectivity and caused significantly higher infection of iPSC-CMs compared to the addition of 2DG concurrent with exposure to iPSC-CMs. **B** and **C.** Glycolysis has impact in both infection and replication rate. **B.** The effects of 2DG, added at different times throughout infected cultures (axis X represents hours post infection when the 2DG was added) and analyzed 24 hpi indicates that glycolysis is important for *T. cruzi* infection and intracellular parasite survival. **C.** The effects of glycolysis on *T. cruzi* replication were assessed by adding 2DG at different points during the first 24 hours (axis X) and analyzed at 48 hours. Parasite’s replication was impaired when 2DG was added at 24 hpi. **D** Rat neonatal cardiomyocyte replicate responses observed in infected iPSC-CMs with and without 2DG treatment. **E.** 2DG and mannose treatment of iPSC-CMs did not recover normal levels of parasite infection and replication, indicating that treatment did not substantially impact protein N-glycosylation. **F.** Nonspecific inhibition of GLUT isoforms by quercetin abrogated both infection and replication. Data reflects analyses of three independent replicate samples for each experiment. **G.** Fluorescent images of *T. cruzi* – infected iPSC-CMs treated with and without 2DG. GFP-tagged parasites, green; host and parasite DAPI-stained nuclei, grey; alexa-555 phalloidin-stained actin cytoskeleton, red. Statistical analyses used ANOVA test with Bonferroni correction and p-value<0.05 was considered significant.

**S9 Fig**


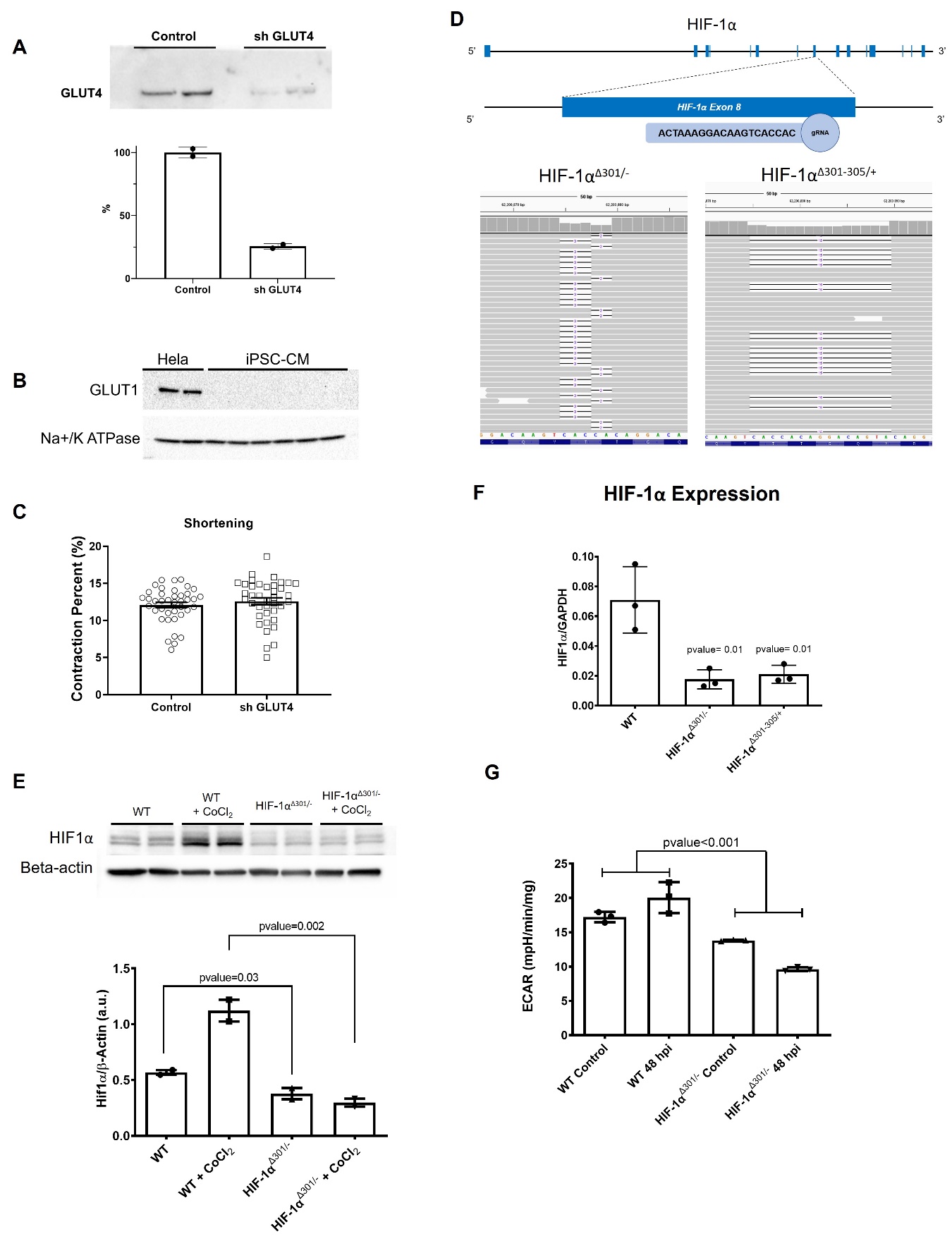


**S9 Fig**. **A.** iPSC-CMs transfected with short hairpin RNAs targeting GLUT4 reduced the membrane expression of these glucose transporters. **B.** iPSC-CMs showed undetectable levels of GLUT1 in membrane. HeLa was used as positive control and anti-Na+/K ATPase was used as loading control. **C.** iPSC-CMs transfected with short hairpin RNAs targeting GLUT4 did not alter contractile function and viability. **D.** Generation HIF-1α mutant iPSC-CMs. iPSCs transfected with gRNA targeting exon 8 sequences in HIF-1α were cloned and genomic sequences studied using an Illumina MiSeq. The sequences of targeted clones studied in this manuscript are visualized using Integrated Genomics Viewer (IGV). HIF-1α^∆301/-^ denotes a compound inframe deletion of amino acid residue 301 (T) and a premature stop codon. HIF-1α^∆301-305/+^ denotes a heterozygous inframe deletion of amino acids residues 301- 305 (TTGQY). **E.** Protein levels of WT and HIF-1α mutant iPSC-CMs. HIF-1α levels in mutant lines did not increase in response to induction with hypoxia using cobalt chloride (CoCl_2_). **F.** HIF-1α gene expression in WT and mutant lines. **G.** Extracellular acidification rates (ECAR) are consistent with reduced glycolysis HIF-1α mutant iPSC-CMs cat baseline and after *T. cruzi* infection. Statistical analyses used T-test and p-value<0.05 was considered significant. Data reflects analyses of two independent replicate samples for each experiment in A and E, and three independent replicate samples for each experiment in F and G.
